# Exploring the therapeutic mechanisms of millet in obesity through molecular docking, pharmacokinetics, and dynamic simulation

**DOI:** 10.3389/fnut.2024.1453819

**Published:** 2024-10-18

**Authors:** Komal G. Lakhani, Rasmeih Hamid, Sheetal Gupta, Poojaben Prajapati, Ratna Prabha, Saumya Patel, Kirankumar P. Suthar

**Affiliations:** ^1^Department of Plant Molecular Biology and Biotechnology, N. M. College of Agriculture, Navsari Agricultural University, Navsari, India; ^2^Department of Plant Breeding, Cotton Research Institute of Iran (CRII), Agricultural Research, Education, and Extension Organization (AREEO), Gorgan, Iran; ^3^Department of Genetics and Plant Breeding, Navsari Agricultural University, Navsari, India; ^4^Department of Botany, Bioinformatics, and Climate Change Impacts Management, School of Sciences, Gujarat University, Ahmedabad, India; ^5^ICAR-Indian Agricultural Research Institute, New Delhi, India

**Keywords:** obesity, human fat mass and obesity associated protein (FTO), little millet, molecular docking and molecular dynamics (MD) simulation, ADMET, binding free energy

## Abstract

Obesity, a prevalent global health concern, is characterized by excessive fat accumulation, which confers significant nutritional and health risks, including a shortened lifespan and diminished wellbeing. Central to the regulation of energy balance and food intake is the fat mass and obesity-associated (FTO) protein, which modulates the interplay between caloric consumption and energy expenditure. Given its pivotal role in obesity regulation, the identification of effective inhibitors targeting the FTO protein is imperative for developing therapeutic interventions. Currently available anti-obesity drugs are often plagued by undesirable side effects. In contrast, natural plant-derived bioactive compounds are gaining prominence in the pharmaceutical industry due to their efficacy and lower incidence of adverse effects. Little Millet, a traditional cereal known for its rich nutritional profile and high satiety index, was investigated in this study using molecular docking and dynamics simulation approach for its potential as an anti-obesity agent. Our research demonstrates that four bioactive compounds from Little Millet exhibit superior binding energies ranging from 7.22 to 8.83 kcal/mol, compared to the standard anti-obesity drug, orlistat, which has a binding energy of 5.96 kcal/mol. These compounds fulfilled all drug-like criteria, including the Lipinski, Ghose, Veber, Egan, and Muegge rules, and exhibited favorable profiles in terms of distribution, metabolism, and prolonged half-life without toxicity. Conversely, orlistat was associated with hepatotoxicity, a reduced half-life, and multiple violations of drug-likeness parameters, undermining its efficacy. Molecular dynamics simulations and Gibbs free energy assessments revealed that the four identified compounds maintain stable interactions with key residues in the FTO protein’s active site. We propose further validation through extensive *In vitro*, *In vivo*, and clinical studies to ascertain the therapeutic potential of these compounds in combating obesity.

## 1 Introduction

Obesity is primarily characterized by an excessive accumulation of body fat (1). In today’s society, due to lack of exercise and excessive calorie consumption, there has been a remarkable increase in fat storage, leading to notable consequences such as hypertension, diabetes, coronary heart disease and osteoarthritis (2). Around the world, obesity is a major health problem caused by the deposition of fat in adipose tissue, leading to a body mass index (BMI) of 30 kg/m^2^ or more (3). According to the latest World Health Organization statistics, more than one billion people worldwide suffer from obesity and this number is expected to increase by 167 million by 2025. The accumulation of fat has increased considerably in modern times. This is due to inadequate exercise, genetic predisposition and excessive calorie intake through the consumption of sugary and fatty junk food or overeating, incorrect timing of meals and lack of physical activity (4–6).

This leads to several serious health problems such as diabetes (7), hyperinsulinemia (8), cancer (9), heart problems (10), hypertension (11), hyperlipidemia (12) and joint problems (13). Interest in dietary and complementary approaches for the treatment of obesity and related metabolic disorders has grown due to the complexity of pharmaceutical therapies such as sibutramine, rimonabant, orlistat, fenfluramine and phentermine, which have been discontinued due to various side effects (14). Alternative treatments utilizing natural sources have emerged as possible alternatives with minimal or no negative consequences (15). Nowadays, more emphasis is placed on focused diet and consumption patterns (16).

Foods made from cereal grains play an important role in the diet of most people, providing them with significant amounts of energy and essential nutrients (17, 18). Little Millets (*Panicum sumatrense* Roth. ex Roem. & Schult) are small-seeded cereals belonging to the Poaceae family and are a staple food among tribal populations. They offer substantial nutritional value due to their high content of minerals and bioactive compounds, such as phenols, flavonoids, and polyunsaturated fatty acids (PUFAs). Notably, Little Millet is beneficial for weight management owing to its low glycemic index, which contributes to fat reduction in the body (19).

A high prevalence of non-communicable diseases, including obesity, has been linked to insufficient dietary fiber intake and high glycemic index diets (20). Little Millet’s high fiber composition and low glycemic index not only enhance gastrointestinal motility but also significantly lower postprandial glucose and serum cholesterol levels. This contributes to a reduced risk of obesity and associated metabolic disorders. Specifically, Little Millet (*Panicum sumatrense*) contains 3–3.5 times more fiber than staple cereals such as wheat, rice, and maize, establishing it as a superior candidate for promoting health through dietary interventions (21).

Furthermore, evidence suggests that millet-based diets foster prolonged satiety, thereby reducing overeating tendencies due to delayed hunger onset attributed to high dietary fiber content. One study reported a mean weight reduction of 1.9 kg after regular consumption of mixed millet-based foods rich in fiber (29.5 g) over a 3.8-month period, highlighting the fiber’s role in weight management (22). Additionally, research on foxtail millet (*Setaria italica* L.) has demonstrated a reduction in adiposity and increased secretion of leptin, a hormone crucial for regulating energy balance and satiety (23). Another study showed that continuous consumption of barnyard millet-based foods with an average glycemic index of 59.10 over 28 days contributed to weight reduction in individuals aged 27–50 years (24).

Based on previous promising findings, the present study seeks to explore the interactions between bioactive compounds found in Little Millet and the fat mass and obesity-associated (FTO) protein. The fat mass and obesity-associated protein (FTO), first identified as m6A demethylase, is involved in the deposition of triglycerides in hepatocytes and promotes adipogenesis and obesity (25). It also has an effect on appetite suppression, energy metabolism, homeostasis and energy balance (26). In the fight against obesity, the activation of anorexigenic hormones is a successful approach (14). By acting as appetite suppressants, anorectic drugs cause a reduction in calorie consumption, which ultimately leads to a shortage of food and consequently to weight loss. Understanding the link between Little Millet and obesity is crucial for implementing effective strategies to combat obesity. The consumption of small millets including Little Millet as a functional food is steadily increasing. Therefore, this study was conducted to identify specific interactions between Little Millet molecules that bind to FTO using *in silico* approaches. In addition, absorption, distribution, metabolism, excretion, and toxicity (ADMET) profiling and stimulated dynamics of selected peptides were performed to investigate the ligands that fulfill all these requirements and can be identified as potential anti-obesity agents.

## 2 Materials and methods

### 2.1 GC–MS analysis

Gas chromatography–mass spectrometry (GC–MS) was used to elucidate the metabolite profile of dwarf millet (*Panicum sumatrense* L.) grains using a method adapted from Li et al. (27) with some modifications to optimize standardization (27). First, 100 mg of dried, powdered seeds of small millet were frozen in liquid nitrogen and then crushed in 1000 μL of ice-cold 70% methanol (GC–MS grade). The methanolic extract was then transferred to 5 mL Oak Ridge centrifuge tubes (Tarson, USA), shaken for 2 min and incubated in a water bath at 65°C for 10–15 min. After cooling to room temperature, the extract was incubated overnight at 4°C. The next day, 700 μL of chloroform was added to the extract, mixed by pipetting and 1,000 μL of pure deionized Millipore water was added. The mixture was centrifuged at 10,000 rpm for 8–10 min, resulting in separation of the extract into polar and non-polar phases. Each phase was then transferred to new 2 mL tubes and dried under vacuum. The dried phases were derivatised by adding 100 μL of MSTFA [N-methyl-N-(trimethylsilyl)-trifluoroacetamide], followed by incubation in a shaker at 37°C for 60 min with continuous vigorous shaking. The derivatised polar and non-polar phases were combined in equal parts, filtered through a 0.22 μm syringe filter to remove any residues and analysed using an Agilent 7890A GC–MS instrument (Agilent, USA). A 1 μL aliquot of the sample was injected with a split ratio of 25:1 and a helium flow rate of 1 mL/min. The oven temperature was initially set to 250°C for 2 min, then increased to 50°C and held at 300°C for 15 min. After a 7-min solvent delay, the 5975C Inert XL MSD detector operated in scan mode with electron impact ionization (70 eV) over a mass-to-charge ratio (m/z) range of 50–800.

### 2.2 Molecular docking

#### 2.2.1 Protein preparation

The three-dimensional structure of the target protein, i.e., fat mass and obesity associated (FTO) protein (PDB ID: 4IDZ) was retrieved from the RCSB protein database (28).^1^ The structure of FTO in *Homo sapiens* had a chain A of 495 amino acids and a resolution of 2.46 Å (Table 1). Protein preparation was performed using YASARA Structure software (version 19.12.14) through the following steps: (a) arrangement of bond(s), (b) addition of Kolman charges, (c) calculation of Gasteiger charge, (d) elimination of heteroatom coordinate and water molecule, (e) restoration of missing c-terminal oxygen, and (f) addition of hydrogen atoms The optimisation process also prioritized the improvement of hydrogen bonds while minimizing other factors, using AMBER14 for the field after removal of hetero groups. Assessment and verification of protein quality and the specific region where the protein binds with the ligand was performed using the SiteMap application and saved as a .pdbqt file.

**Table 1:** Characteristics of target protein 4IDZ to inhibit obesity.

PDB ID	4IDZ
Structure	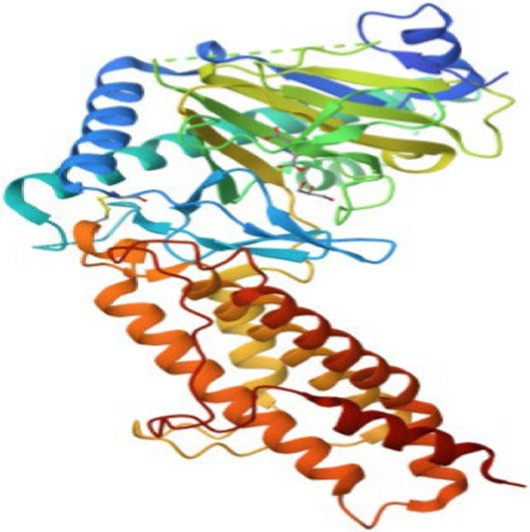
Resolution	2.46 Å
R-value free	0.245
R-value work	0.194

#### 2.2.2 Preparation of the ligands

A total of 133 bioactive compounds were identified from the methanolic extract of Little Millet through GCMS. And were used as ligands for molecular docking. The 3D structure of all compounds was obtained from the PubChem database.^2^ Ligand preparation was performed using Maestro software (29), which provides various functions such as atom and bond queries, stereochemistry, customized templates and valence detection. In addition, charges were added, twist angles were calculated and the energy was minimized at a gradient of 0.05. The steepest gradient method was used to optimize the geometry for a total of 100 iterations.

#### 2.2.3 Virtual screening

Virtual screening of ligands for bioactive compounds of Little Millet was performed with YASARA to identify binding sites for target proteins (30). The crystallized ligand coordinates were processed with the “Clean” module, and the AMBER04 force field determined atom types to confirm vina generated docking sites similar to their original position. A high positive score indicates strong binding between the ligand and its target receptor, in contrast to conventional scoring functions where a negative score indicates better binding between them (31). The ACCELRYS Discovery Studio (32) was used to visualize the natural products selected based on the enumerated intermolecular interactions using the AMBER4 force field (33). The compounds that showed the best interaction with the receptor and the highest binding energy were selected for further investigation using molecular dynamics (34).

### 2.3 *In silico* physico-chemical and ADMET studies

Before an active substance is optimized and developed as a drug candidate, the properties of ADMET and other pharmacokinetic parameters must be thoroughly investigated (35). Different online web servers, i.e., SwissADMET (36), pkCSM (37), and ADMETlab were used for screening process to filter out the top three compounds.

### 2.4 Molecular dynamics simulation

Following the results of the molecular docking, the most promising compounds identified for their efficacy in inhibiting receptor proteins were selected for further analysis using molecular dynamics (MD) simulations. These simulations evaluated ligand-protein complex stability, atomic motions, large conformational changes and ligand binding efficacy using the Desmond package in Schrödinger software (Schrödinger Release 2020–3), with a simulation time of 100 ns per run (30). Prior to the simulations, Maestro software was used to ensure that the three-dimensional (3D) structures of the ligands remained intact and no conformational changes occurred due to the addition of hydrogen bonds. The Protein Preparation Wizard was used to prepare the ligand-protein complexes so that the complexes could be relaxed prior to the simulations. The OPLS all-atom force field (2005) in an orthorhombic simulation box with the dimensions 10 Å × 10 Å × 10 Å was used for the simulations. Energy minimization was performed using the steepest descent method, with the OPLS2005 force field and heating from 0 to 300 K (38). The dynamics simulation was performed at constant pressure (1.01325 atm) and constant temperature (10 K) for 1 μs (39). To maintain charge equilibrium and avoid artifact forces, counterions (Na^+^ and Cl^−^) were added to neutralize the system. The stability and structural integrity of the *Panicum sumatrense* L. (Little Millet) derived ligands complexed with the FTO protein and compared with orlistat were evaluated by monitoring key parameters such as protein-ligand contacts, 2D structural changes in the protein, radius of gyration, molecular surface area, polar accessible surface area, root mean square deviation (RMSD), root mean square fluctuation (RMSF) and torsional profiles using the Simulation Interaction Diagram wizard tool.

### 2.5 Binding free energy calculation

The protein–ligand complexes of the first four top compounds which act as the strongest inhibitors of the FTO protein, were identified by virtual screening. The free energy of binding (ΔG) was calculated using the Schrödinger suite by the Molecular Mechanics Poisson-Boltzmann Surface Area (MM/GBSA) approach. The free solvation energy were considered to understand the effects of individual amino acids and how they change over time (15). A more negative value means a stronger binding energy. Ultimately, the free energy of binding of each protein-ligand complex was determined by selecting only the stable last 20 nanosecond trajectories from the molecular dynamics simulation. The following equation was used to calculate the binding free energy:

ΔGbind = ΔGcomplex – (ΔGprotein + ΔGligand).

## 3 Results and discussion

### 3.1 Identification of bioactive compounds from little millet using GC–MS

The main objective of the GC–MS analysis was to achieve a comprehensive identification and quantification of the phytoconstituents present in the dwarf millet grains. Given the complex physiological and metabolic processes in crop plants, metabolomics provides a valuable approach for the identification of key genes for nutrition by focusing on basic metabolic aspects (40). One of the most important applications of metabolomics is the generation of nutrient and metabolite profiles for different varieties of Little Millet, facilitating the creation of digital markers for rapid identification of the different traits of these seeds. Such tools are of great importance for the development of biomarkers related to nutritional and physiological traits.

Little Millet, which belongs to the minor millets group, is often referred to as a “superfood” due to its numerous health-promoting properties (41). In particular, Little Millet, an important cereal, has a high content of unsaturated fatty acids especially oleic acid (868 mg) and linoleic acid (1,230 mg) and contains no trans fats (0%) (42). Its grain, which is characterized by a low glycaemic index and high fiber content, promotes satiety, aids digestion and reduces overall calorie intake, making it an ideal dietary ingredient to support metabolism and promote healthy weight management (43). As it also contains no gluten, millet is also suitable for people with coeliac disease. Despite its significant nutritional properties, Little millets have received relatively little attention compared to other staple cereals.

In this study, GC–MS analysis identified 133 untargeted phyto-constituents in Little Millet, as listed in Supplementary Table 1. The bioactive compounds identified include phenols (e.g., catechin, ferulic acid, caffeic acid, gallic acid), alkaloids (e.g., quercetin, procyanidin B1, atropine), fatty acids (e.g., 9,12-octadecadienoic acid, n-hexadecanoic acid, erucic acid), flavonoids (e.g., naringenin, luteolin), sugar acids (e.g., D-gluconic acid, D-ribonic acid), sugar alcohols (e.g., D-glucitol, myo-inositol) and organic acids (e.g., butanoic acid, propanoic acid). Several of these compounds, such as 9-octadecenoic acid, quercetin, naringenin and catechin, have been shown to support weight management by reducing inflammation and improving fat metabolism. Importantly, these bioactive compounds, which are not normally found in staple cereals like wheat and rice, position Little Millet as a functional food with significant potential to improve human health. This study provides the first scientific report on the identification of bioactive compounds in Little Millet that could serve as safer and more effective alternatives to treat obesity by promoting fat metabolism and reducing fat accumulation. As a result, this research increases the nutritional value and global appeal of Little millet and advocates its increased consumption as part of a healthy diet.

### 3.2 Binding modes and molecular interactions

**Figure 1:**
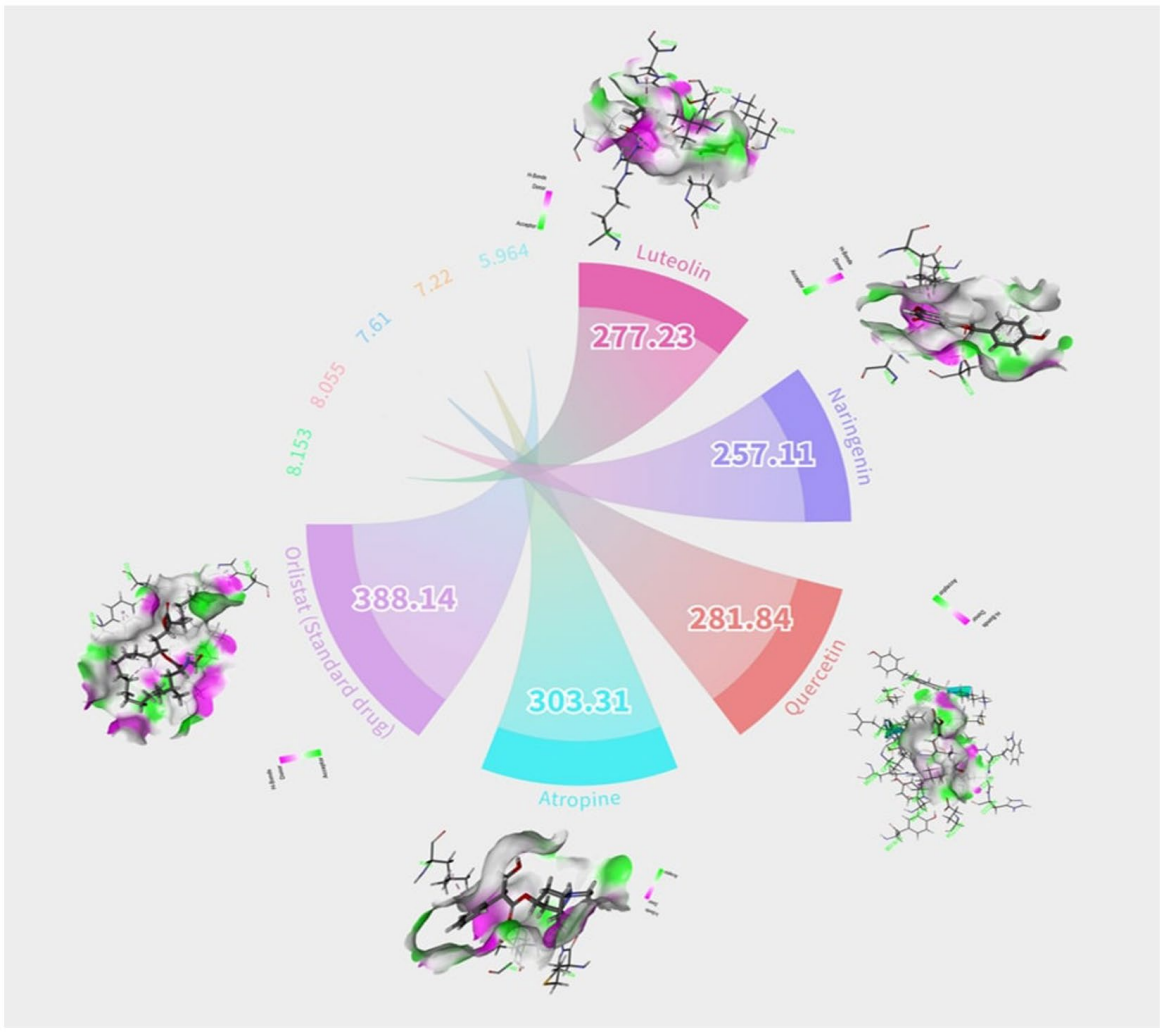
Graphic/Circos representation of potential bioactive compounds in little millet decipited with their hydrogen bonds and binding energy.

The identification of anti-obesity inhibitors from Little millet phytochemical compounds against the target protein 4IDZ by virtual screening based on molecular docking and molecular dynamics simulation provides valuable insights into their therapeutic potential. In this study, a key compound from small millet was docked to the receptor protein using a blind docking approach, which allowed a comprehensive understanding of the binding of the reference inhibitor to the active site. This method enabled the prediction of the most promising active compounds by investigating potential binding interactions at all sites of the protein and facilitated the identification of different types of inhibition, including competitive, non-competitive and mixed inhibition (44, 45). Of the 133 phytochemicals identified, four compounds — luteolin, naringenin, quercetin and atropine showed the strongest inhibition of the fat mass and obesity-associated (FTO) protein, with high binding affinity and strong interactions compared to the standard anti-obesity drug, orlistat (Figure 1). Table 2 provides a detailed overview of the intermolecular interactions between these important phytochemicals and orlistat, highlighting their interactions with the amino acids at the active site of the FTO. Of the compounds studied, luteolin exhibited the highest binding energy, indicating its potential as a superior FTO inhibitor and promising anti-obesity agent.

**Table 2:** The calculated binding energy, hydrogen bonds, and contacting receptor residues of co-crystal ligand and top-three natural compounds with alpha amylase (PDB ID: 4IDZ) using YASARA structure.

Ligand	Pubchem ID	Ligand-protein intereaction	Effi [kcal/(mol*Atom)]	Bind.energy (kcal/mol)	Dissoc. constant (pM)00000001059833.8750	Con. Surf (A^2)	Amino acid involved in hydrogen bond
Luteolin	5280445	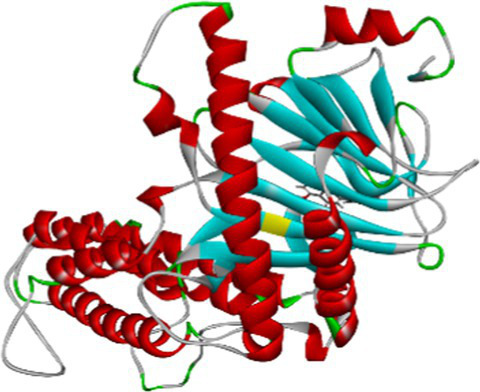	3882	8.1530	1056262.2500	277.23	LEU90, VAL94, THR92, ARG96, TYR108, HIS231, VAL228, LEU109, ILE85, SER229, ALA227, PRO93, MET226, LEU215, LYS216
Naringenin	439246	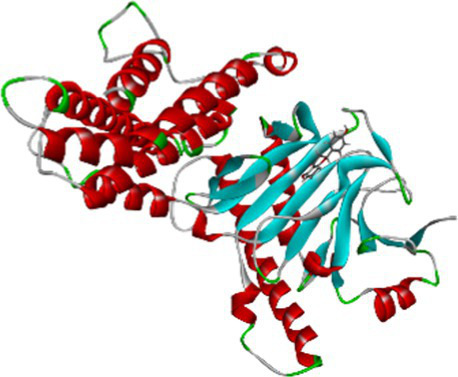	4028	8.0550	1246254.3750	257.11	ARG96, TYR108, HIS231, LEU109, LLE85, SER229, MET226, LYS216, LEU90, PRO93, ALA227, VAL228, THR92, VAL94
Quercetin	5280343	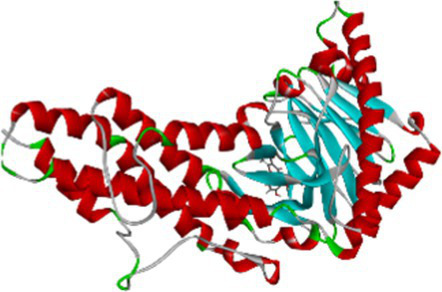	3459	7.61	2641168.0000	281.84	LEU109, VAL228, ILE85, PRO93, LEU90, LYS216, LEU215, MET226, ALA227, THR92, SER229, ARG96, TYR108, GLU234, HIS231
Atropine	174174	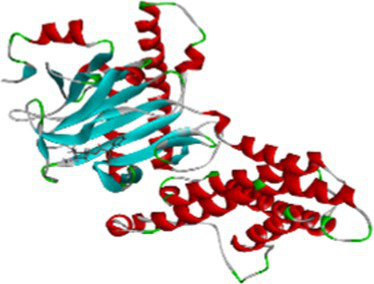	3439	7.22	5083979.5000	303.31	SER229, ILE85, HIS231, GLU234, TYR108, LEU109, ARG96, THR92, VAL83, LEU91, LEU90, LEU215, TYR214, LYS216, ALA227, MET226, VAL228, PRO93
Orlistat (Standard drug)	3034010	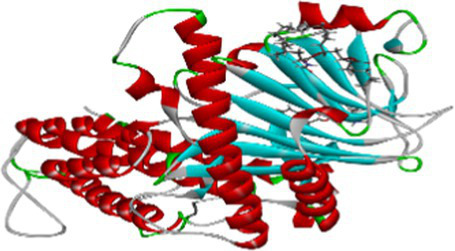	0.1704	5.964	42494236.00	388.14	LEU51, ILE50, TYR39, TRP42, HIS290, PRO288, PRO33, ILE287, ASP293, GLU53, GLN43, PRO47, LYS160, LEU49, ILE50, ARG52, GLU156, PHE38

**Figure 2:**
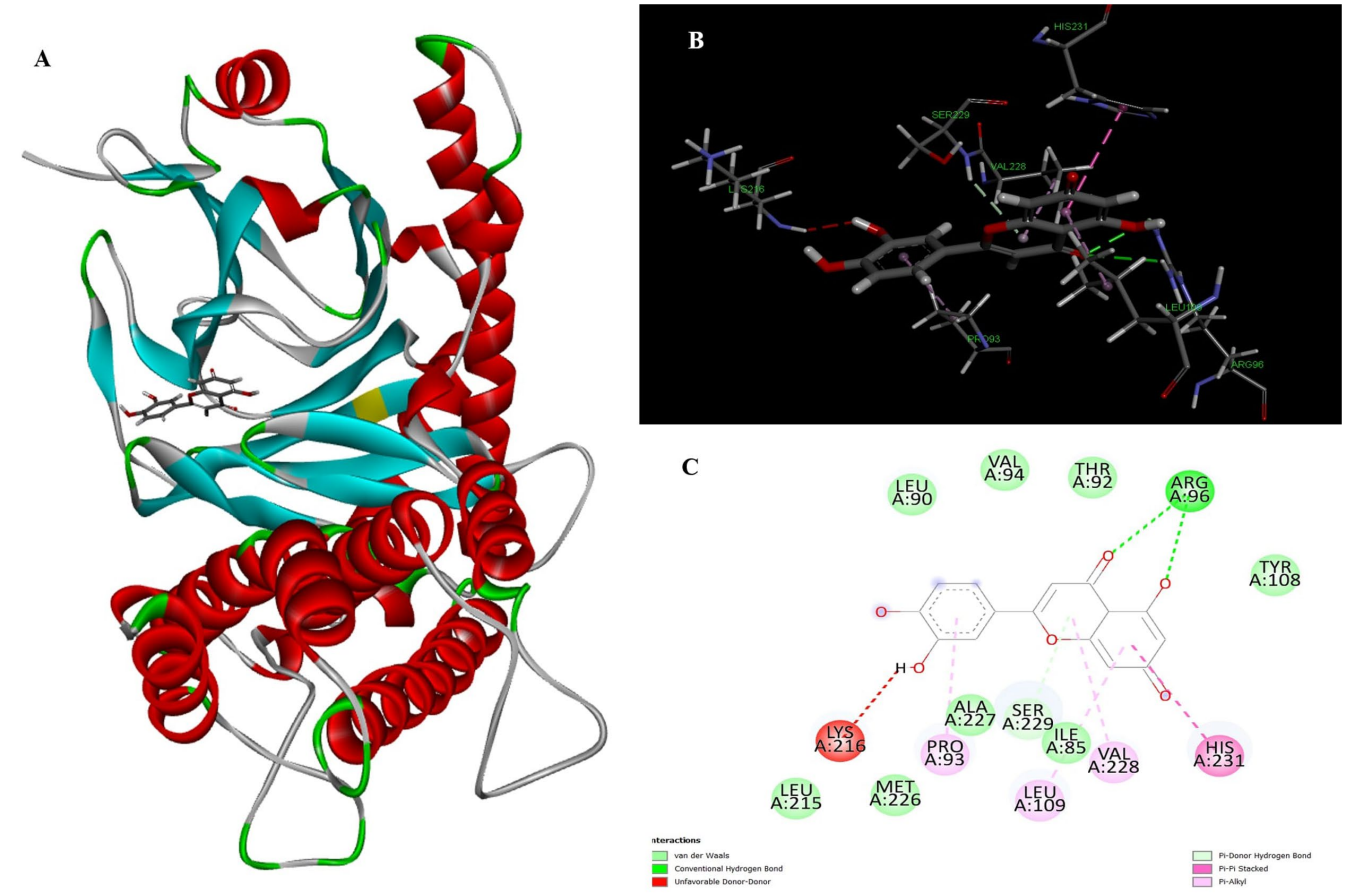
Molecular docking of luteolin to binding site of 4IDZ **(A)** molecular docking complex, **(B)** 3D representation for amino acid residues involved in hydrogen bond donor acceptor binding of luteolin with 4IDZ, **(C)** 2D representation of interaction type of 18,19-Secoyohimban 19-oic acid with surrounding amino acids of 4IDZ.

Luteolin proved to be the most promising compound capable of inhibiting FTO, the protein associated with fat mass and obesity, in the treatment of obesity. Luteolin showed a number of interactions within the binding pocket of the FTO receptor, including two conventional hydrogen bonds, a carbon-hydrogen bond, a pi-pi stacking interaction and several pi-alkyl and van der Waals forces, resulting in a binding energy of −8.15 kcal/mol (Figure 2). Computational analyses revealed that luteolin forms an extensive interaction network at the FTO binding site, in which the Arg96 residue forms two conventional hydrogen bonds with bond lengths of 5.59 Å and 6.32 Å, respectively. In addition, the His231 residue in chain A is involved in a pi-pi stacking interaction with a bond length of 4.48 Å, which contributes to the conformational stability of the protein-ligand complex. The docking results also showed that Ser229 is involved in the formation of a carbon-hydrogen bond and a pi-donor hydrogen bond. Furthermore, pi-alkyl interactions involving His231 (4.48 Å), Pro93 (4.57 Å), Leu109 (4.95 Å), and Val228 (4.45 Å) were observed, stabilizing the active site of the FTO protein and maintaining the energy conformation of the luteolin-bound complex.

**Figure 3:**
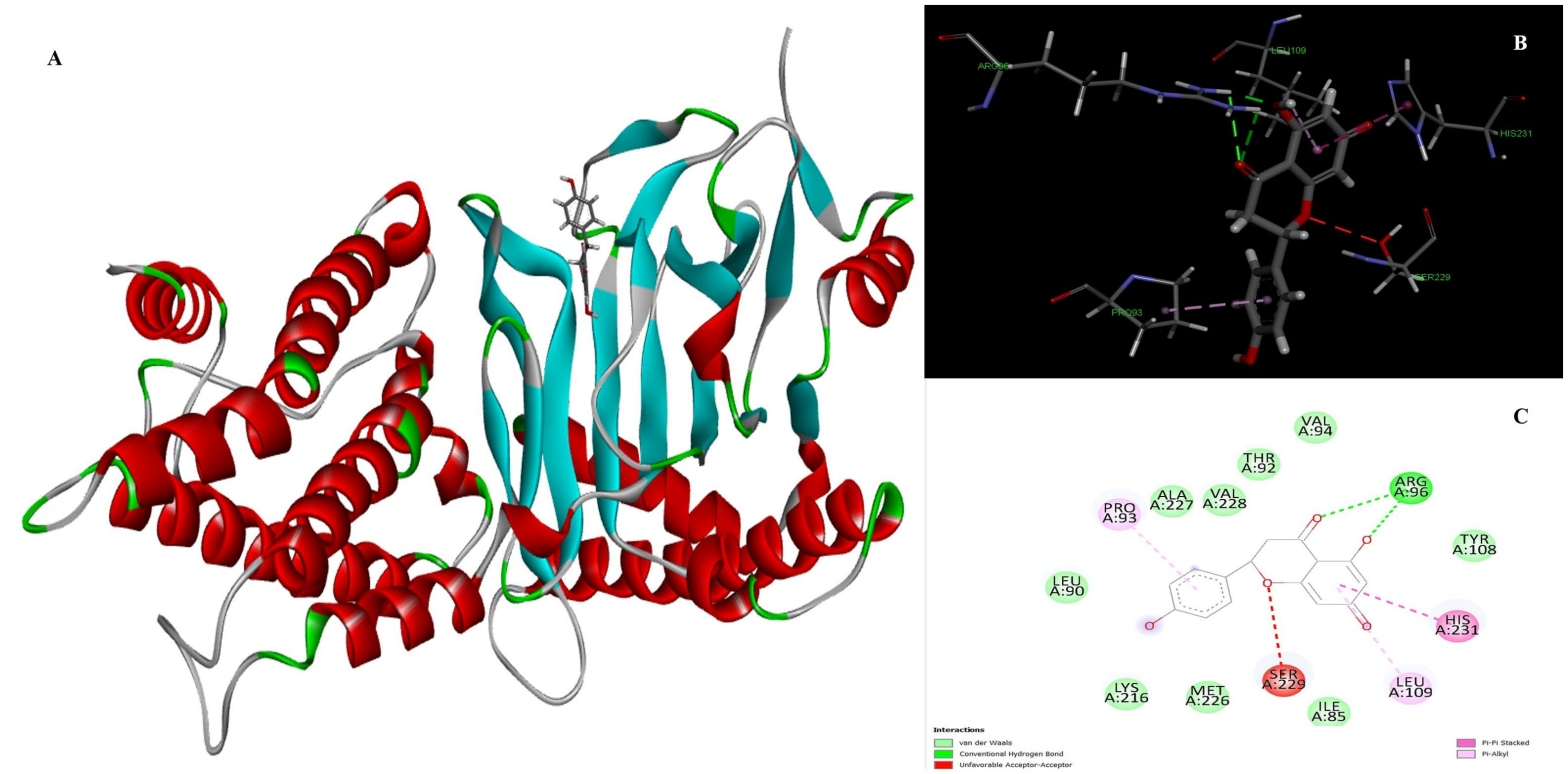
Molecular docking of Naringenin binding site of 4IDZ **(A)** molecular docking complex, **(B)** 3D representation for amino acid residues involved in hydrogen bond donor acceptor binding of naringenin with 4IDZ, **(C)** 2D representation of interaction type of naringenin with surrounding amino acids of 4IDZ.

Naringenin is the second most important compound that can serve as an inhibitor for the treatment of obesity. The stability of complex formation between the target protein and the inhibitor is determined by multiple hydrogen bonds, which have a binding energy of 8.05 kcal/mol in the inhibitors studied. The result shows that, with luteolin, a single amino acid residue Arg96 was involved in forming two conventional hydrogen bonds with a distance of 1.83 Å and 2.74 Å in naringenin. Apart from this, the Pi-Pi stacked and Pi-Alkyl hydrophobic interactions help to maintain the energy and stability of the ligand at the receptor protein docking site (Figure 3). The stacked hydrophobic interaction Pi-Pi formed by His231 (4.01 Å) at a single site was critical for stabilizing the active site of the receptor protein. The non-polar amino acid residues Leu109 and Pro93 with aromatic group bound to the ligand within a radius of 4.96 and 4.23 Å, respectively. These strong hydrophobic properties are responsible for the high affinity and anti-obesity activity of this molecule.

**Figure 4:**
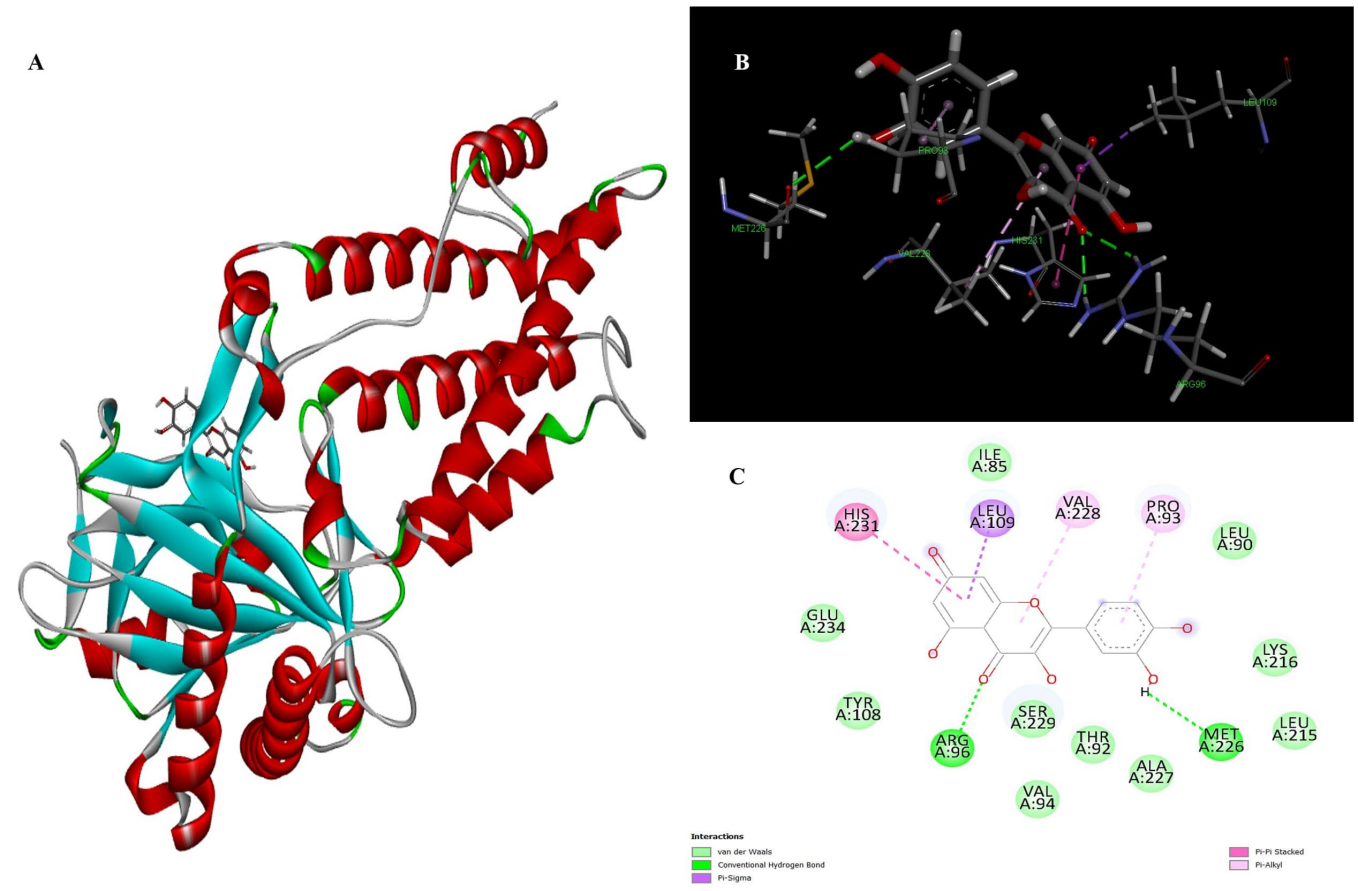
Molecular docking of quercetin binding site of 4IDZ **(A)** molecular docking complex, **(B)** 3D representation for amino acid residues involved in hydrogen bond donor acceptor binding of quercetin with 4IDZ, **(C)** 2D representation of interaction type of quercetin with surrounding amino acids of 4IDZ.

The two-dimensional and three-dimensional shape interactions between the strongest FTO inhibitor quercetin exhibited a binding energy of 7.61 kcal/mol (Figure 4). In addition, the hydroxyl group of the quercetin moiety interacted with Met226 via a conventional hydrogen bond with a bond distance (2.77 Å) and an angle of 103.48. One amino acid residue, Val228 and Pro93, forms two Pi-alkyl interactions with a radius of 4.95 Å and 4.32 Å, respectively, to maintain the energy and stability of the ligand at the receptor protein docking site. An additional Pi – Pi stacked interaction was formed by His231 with the ligand at a binding distance of 4.63 Å. The pi-sigma interaction helps to stabilize the dipole moment of the drug by transferring the charge of the adjacent amino acids. Quercetin interacts via the amino acid residue Leu109 in a binding position of the target protein through a hydrophobic pi-sigma interaction with a bond length of 2.43 Å, which contributes to the stabilization of the drug during intercalation.

**Figure 5:**
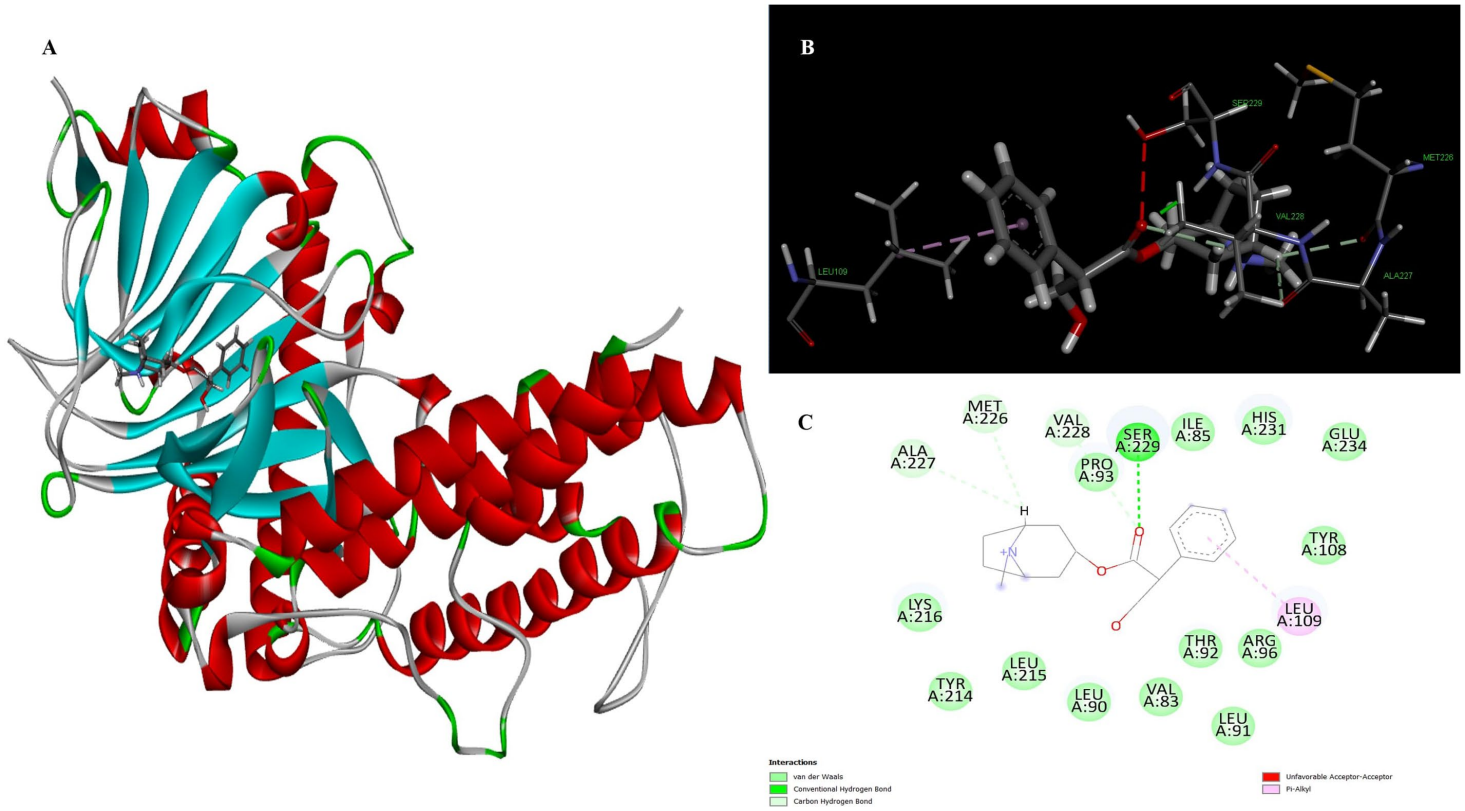
Molecular docking of atropine to binding site of 4IDZ **(A)** molecular docking Complex, **(B)** 3D representation for amino acid residues involved in hydrogen bond donor acceptor binding of atropine with 4IDZ, **(C)** 2D representation of interaction type of atropine with surrounding amino acids of 4IDZ.

Atropine was identified as the fourth most potent potential ligand, exhibiting a binding energy of 7.22 kcal/mol for the FTO protein, which is higher than that of the standard drug orlistat. The docked complex of atropine with the ligand receptor showed stable interactions due to strong conventional hydrogen bonds such as Ser229 at 1.99 Å. In addition, Ala227 (3.02), Met226 (2.83) and Val 228 (2.92) formed carbon-hydrogen bond interactions with the binding pocket of the receptor. Leu109 additionally contributed to a Pi-alkyl interaction with the ligand and exhibited a binding distance of 4.44 Å, effectively reducing the conformational energy of the ligand (Figure 5).

**Figure 6:**
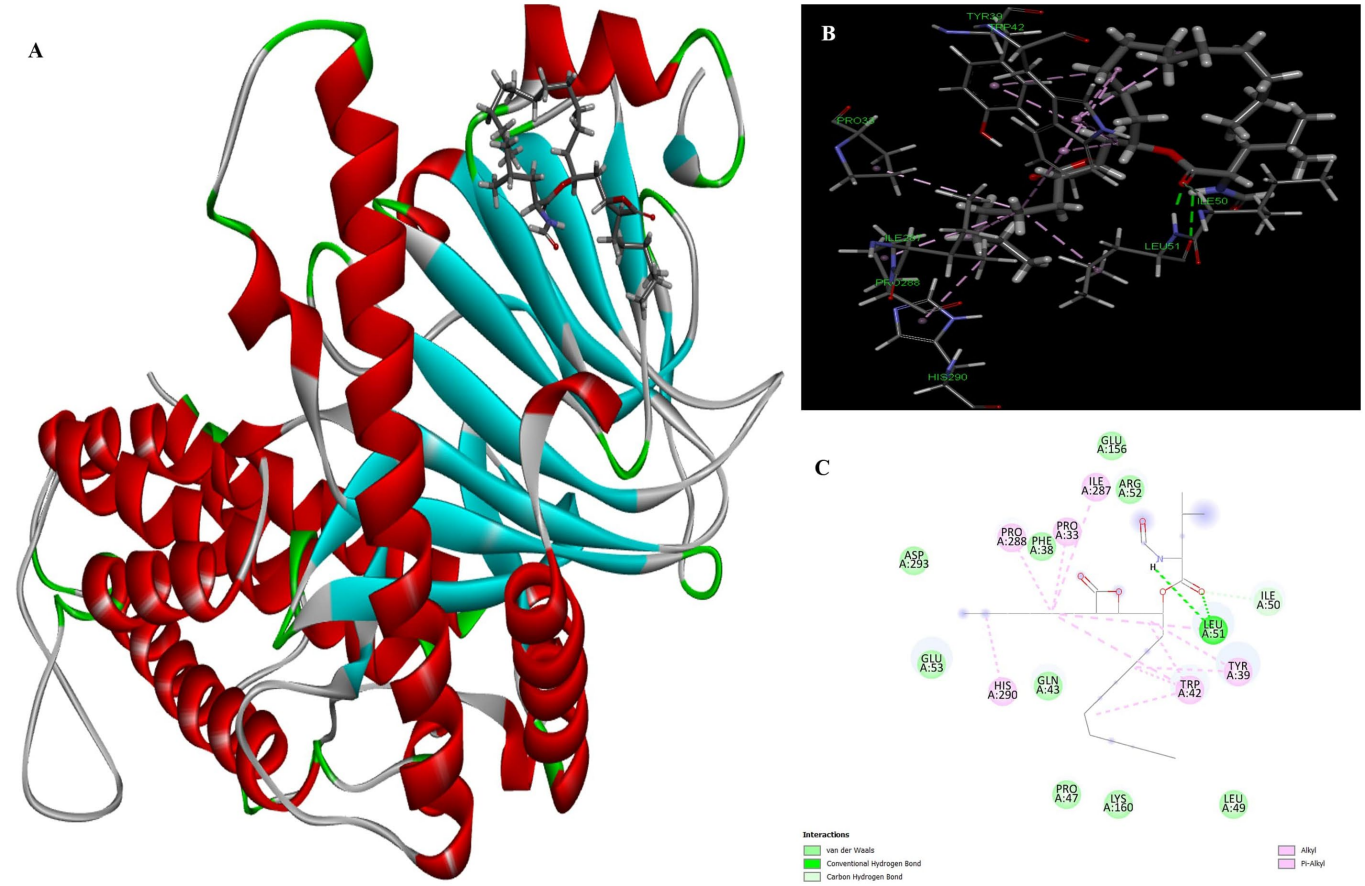
Molecular docking of standard drug orlistat to binding site of 4IDZ **(A)** molecular docking complex, **(B)** 3D representation for amino acid residues involved in hydrogen bond donor acceptor binding of orlistat with 4IDZ, **(C)** 2D representation of interaction type of orlistat with surrounding amino acids of 4IDZ.

The docking result showed that the standard drug orlistat (control) is less effective in the treatment of obesity and binds to the active site of the FTO protein with a binding energy of 5.94 kcal/mol. The 2D interaction of orlistat (Figure 6) revealed that LEU51 forms two conventional hydrogen bonds with distances of 1.98 and 2.99 Å. A carbon-hydrogen bond is formed with -OH of the amino acid residue ILE50 with an angle of 131.99. A total of six amino acid residues, namely Pro288, Pro33, Ile287, His290, Trp42, and Tyr39, are involved in catalysis in the active center of FTO via alkyl and Pi-alkyl interactions. The formation of van der Waal forces between certain amino acids and amide substituents has also been linked to the strong attraction of orlistat. Ultimately, this led to a strong sense of unity and stability within the complex.

### 3.3 *In silico* physico-chemical and ADMET studies

#### 3.3.1 Physicochemical properties and similarity to the drug

The four major compounds extracted from Little Millet seeds were thoroughly evaluated using YASARA to assess their potential as drug candidates, their medicinal properties and their similarity to known lead structures. The use of computational methods in drug discovery has become indispensable as it significantly reduces the need for time-consuming experimental trials while increasing the likelihood of favorable results. Several key parameters are critical in the early stages of drug discovery and development. High-quality drug candidates must have both sufficient efficacy against the therapeutic target and appropriate ADMET properties (absorption, distribution, metabolism, excretion and toxicity) at therapeutic doses. Accordingly, Lipinski’s rule of five was followed, which classifies compounds based on certain criteria and distinguishes between drug-like and non-drug-like substances. For a compound to be considered a viable drug candidate, it must fulfill these criteria (46). The evaluation revealed that all four compounds identified met Lipinski’s rule of five, including molecular weight (MW <500), partition coefficient (MLogP <4.15), number of hydrogen bond acceptors (≤10), number of hydrogen bond donors (≤5) and molar refractivity (MR). In contrast, orlistat did not fulfill one of these criteria, as its MLogP value exceeded 4.15 (47) (Table 3).

**Table 3:** Physicochemical properties of the screened compounds and drugs.

	Luteolin	Naringenin	Quercetin	Atropine	Orlistat
MW (g/mol)	286.24	272.25	302.24	289.37	495.73
NHBAs	6	5	7	5	5
NHBDs	4	3	5	4	1
NRots	1	1	1	1	24
MLog P	1.86	1.75	1.63	2.02	4.66
MR	76.01	71.57	78.03	84.51	145.36
TPSA (Å^2^)	111.13	86.99	131.36	49.77	81.70

Application of the Ghose, Veber, Egan and Muegge filter to evaluate drug likeness yielded promising results, as all preparations matched all five filters, indicating favorable drug-like properties. In contrast, the standard drug orlistat failed to meet at least one of the filters, with three alerts for brenk and lead likeness indicating a lack of drug-likeness and three indicating a lack of drug-like properties (Table 4).

**Table 4:** Predicted drug-likeness, medicinal chemistry and lead-likeness pharmacokinetics parameters of the screened compounds by Swissadmet.

	Luteolin	Naringenin	Quercetin	Atropine	Orlistat
Lipinski #violations	0	0	0	0	1 (MLOGP>4.15)
Ghose #violations	0	0	0	0	4 (MW > 480, WLOGP>5.6, MR > 130, #atoms>70)
Veber #violations	0	0	0	0	1 (Rotors>10)
Egan #violations	0	0	0	0	1 (WLOGP>5.88)
Muegge #violations	0	0	0	0	2 (XLOGP3 > 5, Rotors>15)
Bioavailability score	0.55	0.55	0.55	0.55	0.55
PAINS #alerts	1 catechol_A	0	1 catechol_A	0	0
Brenk #alerts	1 catechol	0	1 catechol	0	3 (aldehyde, four_member_lactones, more than 2 esters)
Leadlikeness #violations	Yes	Yes	Yes	Yes	No 3 violations: MW > 350, Rotors>7, XLOGP3 > 3.5
Synthetic accessibility	3.02	3.01	3.23	4.33	5.42

**Figure 7:**
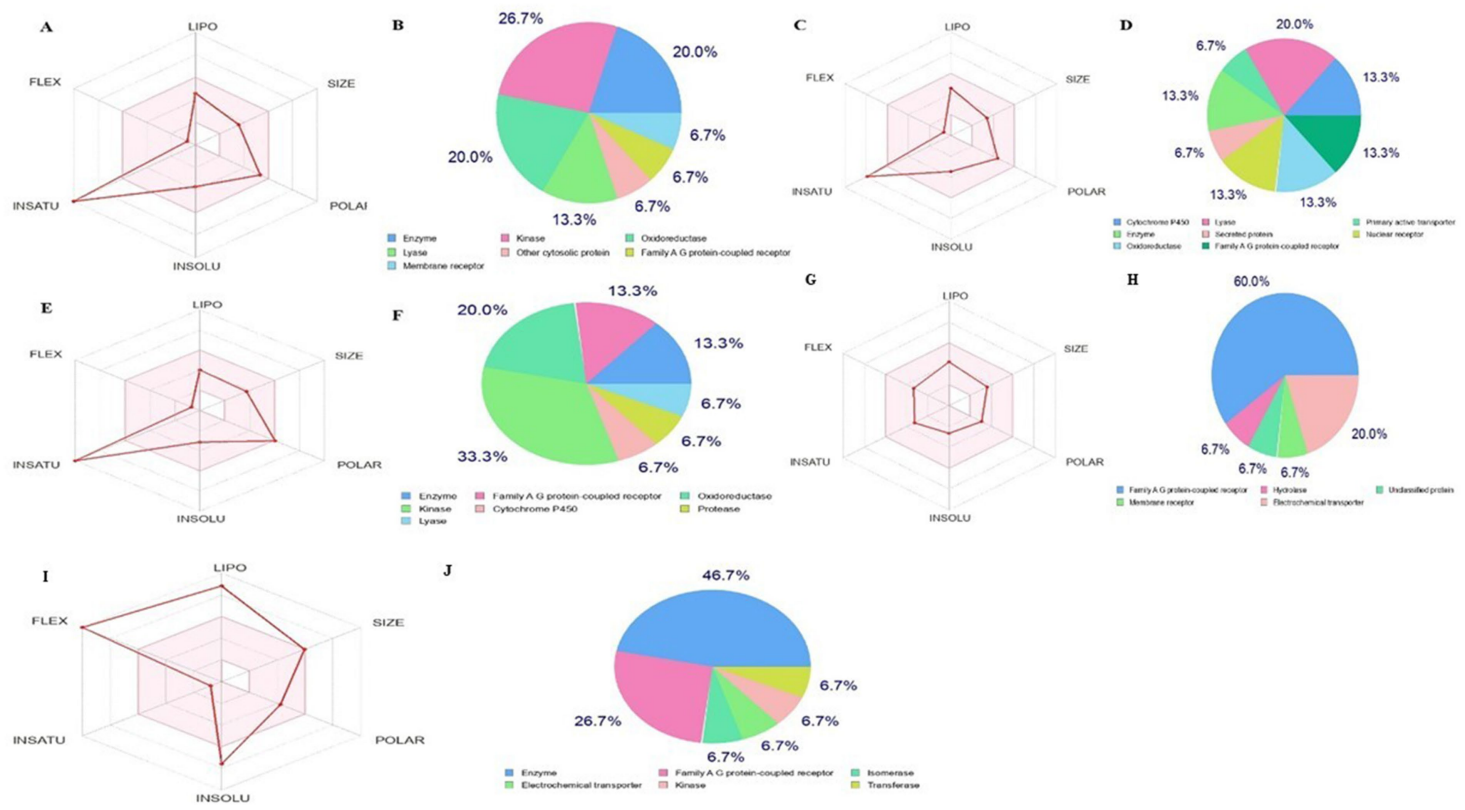
Bioavailibity radar prediction for compounds compounds **(A)** and **(B)** luteolin, **(C)** and **(D)** naringenin, **(E)** and **(F)** quercetin, **(G)** and **(H)** atropine, and **(I)** and **(J)** orlistat using the SwissADME.

The bioactive compounds identified from the Little Millet plant fulfill the parameters for drug, like, oral bioavailability, namely polarity and flexibility, which are determined by physiochemical properties such as the topological polar surface area and the number of rotatable bonds. The metabolic compound is considered to have low oral availability if it has >10 nRt (48) and a TPSA value in the range of 60 to 140 Å^2^, indicating absorption. The bioavailability of the four best analysed hit molecules and orlistat (standard drug) is shown in Figure 7. The result shows that all four bioactive compounds contained in the Little Millet fulfill the requirements for drug properties. In contrast, the standard drug orlistat did not fulfill the flexibility criteria, as the number of rotational bonds (24) is much higher than the desired range. Overall, the bioactive compounds in the Little Millet fulfill all the requirements for a drug with good oral bioavailability compared to orlistat.

#### 3.3.2 Pharmacokinetic properties

For a compound to be effective as an oral drug, it must successfully traverse the intestinal epithelial barrier, a factor that determines its absorption rate and overall bioavailability in the human body. In this study, virtual prediction models are used to evaluate the drug-like properties of different compounds. In modern drug development, an ideal candidate not only exhibits potent efficacy against the target disease but also possesses favorable ADMET (absorption, distribution, metabolism, excretion and toxicity) properties at therapeutic levels.

The pharmacokinetic properties, as calculated using the pkCSM and ADMETSAR web servers, are summarized in Table 5. Based on their molecular characteristics, the findings suggest that oral absorption is generally unfavorable. Nevertheless, atropine exhibited a high absorption rate in the human intestine, with the Human Intestinal Absorption (HIA) value of naringenin closely resembling that of the reference drug, orlistat. The bioavailability and absorption potential of these drug candidates were inversely related to water solubility, Caco-2 permeability and skin permeability. In particular, atropine and naringenin exhibited high Caco-2 permeability, indicating better absorption by the human gut compared to orlistat. In addition, all identified small millet ligands were more likely to cross the blood–brain barrier than orlistat.

**Table 5:** ADMET properties of screened compound and standard drug.

	Luteolin	Naringenin	Quercetin	Atropine	Orlistat
Intestinal absorption (%)	79.391	90.449	77.207	94.508	90.581
Water solubility (log mol/L)	−3.094	−3.212	−2.925	−1.943	−5.293
Caco-2 permeability (log Papp in 10–6 cm/s)	0.27	1.021	−0.229	1.289	0.396
Pgp sub	Yes	Yes	Yes	Yes	No
Pgp In I	No	No	No	No	Yes
Pgp in II	No	No	No	No	Yes
Skin permeability (log Kp)	−2.735	−3.636	−2.735	−4.488	−2.727
PPB (%)	95.436%	93.763%	95.496%	29.684%	97.431%
VD (log L/kg)	−0.985	−0.671	1.559	0.678	−0.547
CYP2D6 substrate	No	No	No	No	No
CYP3A4 substrate	No	No	No	No	Yes
CYP 2C19 Inhibitor	No	No	No	No	No
CYP 2C9 Inhibitor	No	No	No	No	No
CYP 2D6 Inhibitor	No	No	No	No	No
CYP 3A4 In	No	No	No	No	No
T1/2	0.898	0.774	0.929	0.215	0.032
CL	0.554	0.042	0.407	1.013	1.679
Renal OCT 2 Substrate		No	No	No	Yes
AMES toxicity	Yes	Yes	No	No	No
Max. tolerated dose (human) (log mg/kg/day)	1.069	1.06	0.499	−0.044	0.247
Toxicity class	5	4	3	4	4
hERG I inhibitor	No	No	No	No	No
hERG II inhibitor	No	No	No	Yes	No
Oral rat acute Toxicity (LD50) (mol/kg)	2.312	2.208	2.471	2.672	1.972
Oral rate chronic toxicity (log mg/kg_bw/day)	1.981	2.00	2.612	2.358	0.572
Predicted LD50 (mg/kg)	3,919	2000	159	380	1,300
Hepatotoxicity	No	No	No	No	Yes
Skin sensitisation	No	No	No	No	No

P-glycoprotein plays a crucial role in the elimination of harmful substances and toxic xenobiotics from cells. All bioactive compounds, with the exception of orlistat, acted as P-glycoprotein substrates, which could improve the absorption and distribution of drugs in the body. The skin permeability of all compounds was lower than that of orlistat, suggesting a lower dermal toxicity of the investigated compounds.

The distribution and metabolism of drug molecules in the body depend on the volume of distribution and plasma protein binding (PPB). Atropine, in contrast to all other ligands and the standard drug orlistat, showed a lower value for PPB (29.684%) and a higher value for VDss (0.678), suggesting that atropine binds weakly to common blood proteins and distributes more in tissues than in plasma, increasing its efficacy as a drug. The PPB value of orlistat is the highest of all, which plays an important role in reducing its pharmacological effect.

After absorption, the chemical compounds are metabolized in the liver, where cytochrome P450 isoenzymes are active. Despite varying drug concentrations in the organism, the concentration of an enzyme at a particular active site remains constant. Enzymes that are abundant play a crucial role in catalyzing reactions with a low concentration of drugs, applying the principle of a first-order process. Evaluation of an agent as a potential treatment without adverse effects is an important consideration (49). Cytochrome P450 (CYP) or membrane-bound hemoproteins play a critical role in the detoxification of xenobiotics in the liver (50). It is crucial to assess the ability of a compound to interfere with cytochrome P450, as these isoforms can both metabolize and bioactivate various drugs (51, 52). Metabolism analysis revealed that all compounds are non-inhibitors of the P450 isoform, which enhance the metabolism of drugs and prevent the blockade of the binding site of a substrate.

The data on the toxic properties of all selected phytochemicals were compiled and presented in Table 5. The AMES toxicity test is used to determine whether a substance is capable of causing mutations or not (53). There are no reports on the toxicity of the phytochemicals, especially with regard to their mutagenic potential against bacteria (AMES test), they are not hERG-I inhibitors, carcinogenic and do not produce skin sensations. The inhibitors of hERG-II detected by atropine increase the likelihood that they have a negative effect on heart health. The hepatoxicity result showed that all compounds of orlistat are non-hepatoxic. Therefore, the consumption of orlistat does not cause liver damage. In contrast to the potential ligands of Little Millet, the standard drug orlistat has a low LD50 (1.972) and LOAEL (0.572 log mg/kg/day), suggesting that the standard drug orlistat is more toxic and poses a health risk after prolonged consumption.

The excretion of drugs from the body, which is directly related to the bioavailability value of the drug, helps to determine the drug rate. The excretion of drugs occurs through a combination of two different processes, namely hepatic clearance by the liver and renal clearance by the kidney (54). The function and excretion of drugs in the kidney are highly dependent on OCT2, a transporter responsible for the uptake of drugs into the renal system. The result suggests that renal organic transporter 2 (OCT2) is not a substrate for any of the selected phytochemicals that orlistat anticipates (55). All potential ligands identified from Little Millet with potential for the treatment of obesity show clearance values in the range of 0.042–1.013 log ml/min/kg, which is lower than the standard drug orlistat (1.679 log ml/min/kg). This indicates that all potential millet-derived ligands have a longer drug half-life due to a slow clearance rate. In this condition, all potential ligands must be administered less frequently, while orlistat must be administered more frequently to increase therapeutic drug levels. The overall result of the docking and pharmacokinetic properties revealed that the top rated compounds from millet have a higher potential to reduce obesity by inhibiting the activity of fat and associated proteins. In contrast, orlistat was found to violate the rules of drug friendliness, have a low intestinal absorption rate, and be more toxic and may lead to liver damage, reducing its pharmacological effect.

### 3.4 Molecular dynamics simulation

Molecular dynamics simulation is a computational method that can be used to analyse structural changes and the flexibility of docked complexes during a simulation. The top-ranked compounds from the virtual screening allow independent investigation of the conformational stability of the ligand and its binding ability over time in the catalytic pocket of the protein associated with fat mass and obesity. To accurately determine the inhibitory effect of natural products targeting 4IDZ in the binding pocket, it is important to thoroughly evaluate the conformational stability and binding affinity, even if docking provides an approximate arrangement of the ligand in the receptor pocket. Molecular dynamics (MD) simulations provide a thorough investigation of this conformational stability, which is critical for the ligand to successfully inhibit fat mass and obesity-associated protein. Molecular dynamics (MD) simulation trajectories were run for 100 nanoseconds to assess the complex structural and dynamic interactions and stability of the highest binding energy ligand identified from the virtual screening data against the protein associated with fat mass and obesity (15). Before the molecular dynamics simulations were performed, the energy of the solvated systems was minimized. During the simulations, RMSD, RMSF analysis, Rg, SASA, MolSA, and PSA scores of the complexes were analysed.

#### 3.4.1 Root mean square deviation (RMSD)

**Figure 8:**
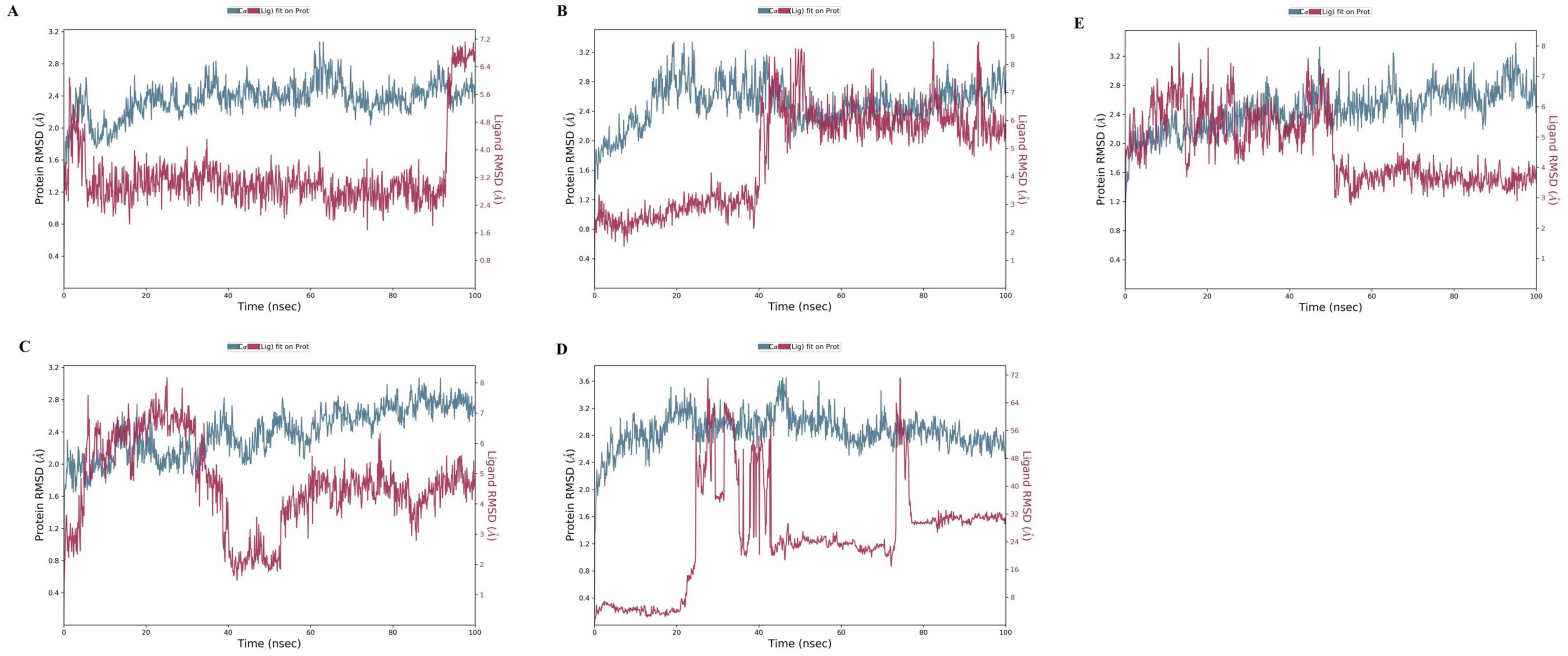
Protein–ligand root-mean-square deviation (RMSD) plot of **(A)** luteolin, **(B)** Naringenin, **(C)** quercetin, **(D)** atropine, and **(E)** orlistat bound to the inhibitory site of 4IDZ protein.

Root Mean Square Deviation (RMSD) is an important metric for assessing the structural variances observed during molecular simulations (56–58). It is a valuable tool for analysing conformational changes in protein structures during simulations, with lower RMSD values indicating greater structural stability. The RMSD plot of the four investigated compounds in complex with 4IDZ (Figure 8) shows that the RMSD values for luteolin, naringenin, quercetin, atropine and orlistat were between 1.6–3.2 Å, 1.8–3.3 Å, 1.7–3.1 Å, 2.0–3.5 Å, and 1.6–3.2 Å, respectively. It is noteworthy that the RMSD values for all compounds remained below 3.5 Å for most of the simulation period.

The ligand RMSD provides information about the stability of the ligand binding within the binding pocket of the protein. The term “lig-fit Prot” refers to the RMSD of the ligand relative to the protein backbone. RMSD values that are slightly greater than those of the protein are normally considered satisfactory. However, significantly larger values may indicate that the ligand occupies a stable position that differs from its original docking site. For the luteolin–4IDZ complex (Figure 8A), the ligand fit prot remains significantly lower than the RMSD of the protein until about 95 ns, when it increases exponentially and surpasses the RMSD of the protein. This behavior indicates that the ligand is likely to remain bound to the original binding site throughout the simulation. In the case of the naringenin–4IDZ complex, the Prot value of the ligand remains lower than that of the protein until about 40 ns with a value of 4 Å. It then increases and stabilizes at around 9 Å up to 100 ns (Figure 8B). Similarly, the quercetin–4IDZ complex shows a slightly higher Lig-Fit-Prot value in relation to the RMSD of the protein up to 40 ns, after which it decreases and stabilizes for the remainder of the simulation (Figure 8C). Of the four compounds, atropine–4IDZ exhibits the greatest fluctuation in RMSD values, suggesting lower stability (Figure 8D). This can be seen from the Lig-Fit Prot values, which fluctuate between 8–72 Å and thus lie significantly above the RMSD range of the protein of 2.0–3.6 Å during the course of the simulation. The RMSD of the reference drug orlistat fluctuates between 3–8 Å, with values in the range of 6–8 Å being observed in the first 45 ns. Subsequently, the RMSD of orlistat decreases to about 4 Å, which is lower than that of the protein and indicates that the ligand may not fit properly into the binding site of the target protein (Figure 8E).

#### 3.4.2 Protein secondary structure analysis

In order to investigate the structural behavior of a protein, a comprehensive understanding of its secondary structure is required. MD simulations can accurately predict secondary structures, even if the simulated structures ultimately do not match the experimental structures. This is because the secondary structures usually assume a rough shape before folding. Accurate identification of the secondary structures can predict the tendency of structure formation, regardless of the significant variations in structural motifs. In the current study, we try to predict the protein structure and not the protein folding, even though the process of secondary structure formation is crucial for understanding protein folding. The orange background highlights the *α*-helical regions, while the blue background highlights the *β*-strand regions. In this study, the plot summarizes the composition of the secondary structure elements for each trajectory at the bottom. Each assignment of residues and secondary structure elements is tracked over time in the graphs. The composition of secondary structure elements for each trajectory is summarized at the bottom of the graph.

**Figure 9:**
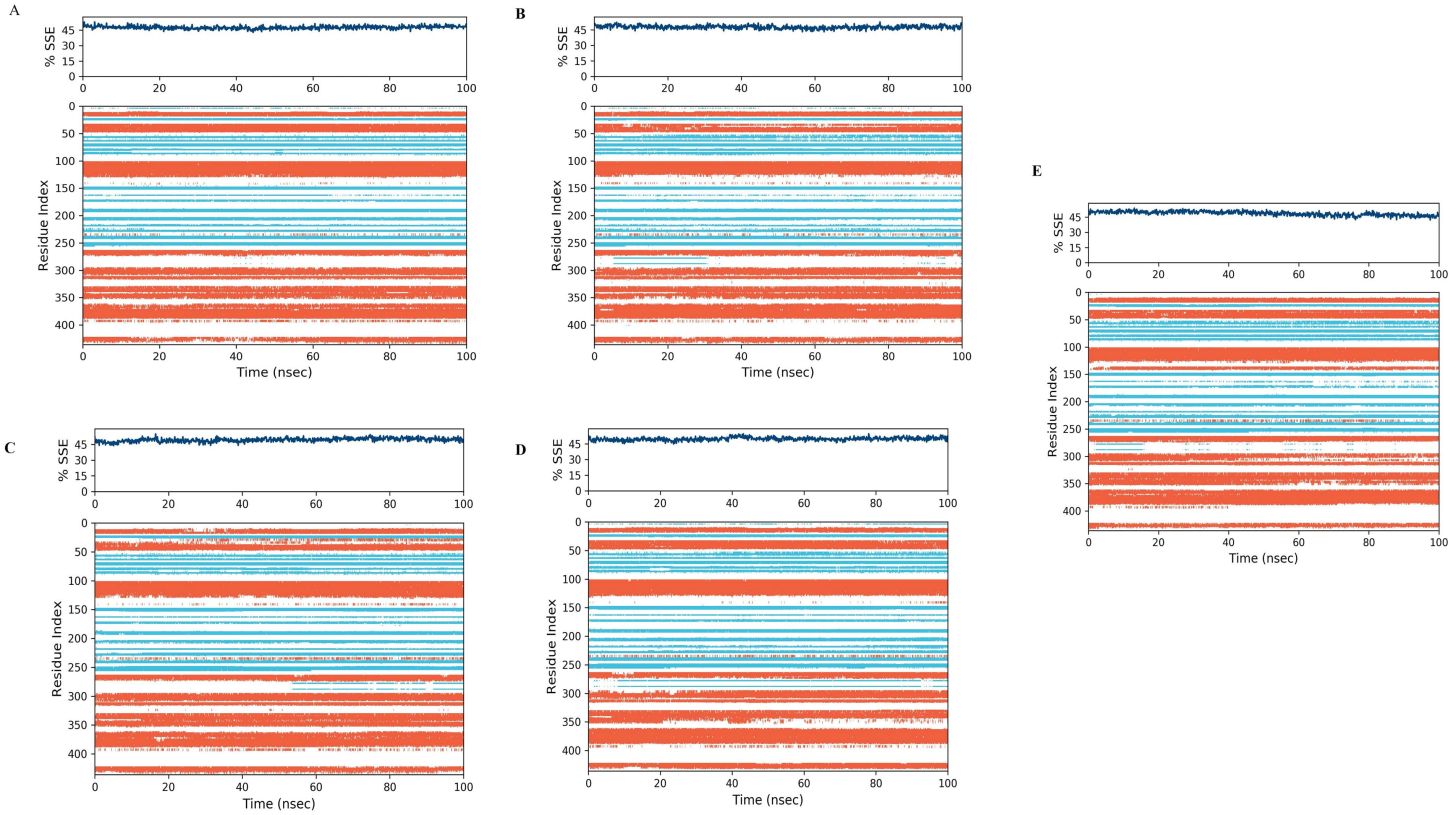
Stability of the 4IDZ secondary structure over 100 ns of MD simulation when complexed with **(A)** luteolin, **(B)** naringenin, **(C)** quercetin, **(D)** atropine, and **(E)** standard drug orlistat. Protein secondary structure elements (SSE) like alpha-helices (orange color) and beta-strands (light blue color) were monitored during the simulation.

The effect of four tested compounds on the α-helix and β-strand regions of the anti-obesity protein 4IDZ was investigated (Figure 9). These regions differed by helices or strands for more than 70% of the simulation time. The secondary structure element composition of luteolin showed a helix and strand composition of 32.16 and 15.51%, respectively, with minor variations observed (Figure 9A). For naringenin, Figure 9B displayed no significant structural changes were observed with 16.34% strand and 31.31% helix, although there were some minor fluctuations. For quercetin, the Figure 9C indicated composition of the helix was 32.55% and the strand was 16.62%. During simulation, SSE analysis of atropine was observed to be 49.73% with 31.58% helix and 18.15% strand element (Figure 9D). It was observed that the ligand remains bound to the active site without any change, indicating that it is incredibly stable. According to the secondary structure analysis, there are no significant differences in the overall conformation of the complex. However, orlistat shows more fluctuation in all strands and helices compared to the four best studied compounds (Figure 9E). The value of total secondary structure elements is 48.66% with 32.05% alpha-helical elements and 16.62% beta-strands. Fluctuations were observed at residue indices 60, 90, 140, 280, 290, 300 and 390, respectively. The instability of the conformation of the protein upon exposure to orlistat may have caused the disruption of the hydrogen bonding structure in the 4IDZ residues, leading to the changes.

#### 3.4.3 Protein–ligand contacts

During the simulation, various interactions between the protein and the ligand were observed. These interactions can be categorized into four main types: water bonds, hydrophobic interactions, ionic bonds and hydrogen bonds. The Simulation Interactions Diagram (SID) board can be used to analyse the subtypes of each interaction type. Non-bonding interactions such as hydrogen bonds allow the interaction of key residues with the binding site and provide information about the activity and binding affinity of the ligand. In addition, analysing the total number of hydrogen bonds formed during the simulation can provide information about the conformational stability of the complex.

**Figure 10:**
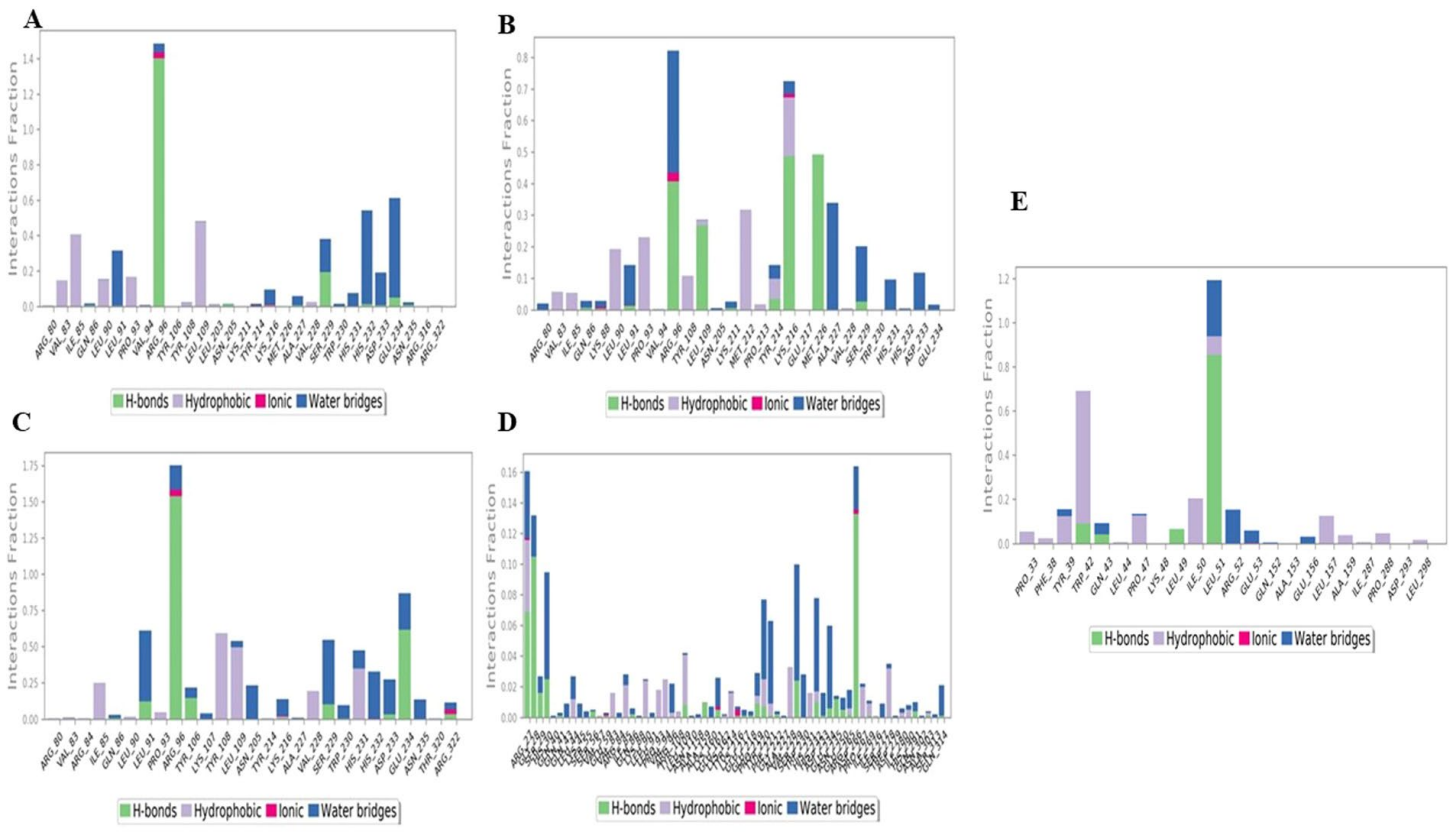
Histogram of the protein–ligand complex of through hydrogen bonds, hydrophobic bonds, ionic bonds, and water bridges exhibited by **(A)** luteolin, **(B)** naringenin, **(C)** quercetin, **(D)** atropine, and **(E)** standard drug orlistat to 4IDZ protein.

In the interaction pattern between the luteolin ligand and the 4IDZ protein complex, hydrogen bonds of eleven amino acid residues were formed: Ile85, Leu91, Arg96, Asn205, Tyr214, Ala227, Ser229, His232, Asp233, Glu234, and Asn235 (Figure 10A). Among these residues, Arg96 had the highest interaction fraction (1.4), followed by Ser229 (0.2), indicating that these two residues contribute significantly to the stability of the complex. In addition, the amino acid residues formed 3 ionic bonds, 15 water bridges and 8 hydrophobic bonds with interaction fractions of up to 0.5, 0.6 and 1.4, respectively. Among these bonds, the fewest amino acid residues were involved in the ionic interactions.

The protein- ligand interaction of the naringenin-4IDZ complex (Figure 10B) revealed that the complex formed a total of 10 HBs through amino acid residues Gln86, Lys88, Leu91, Arg96, Leu109, Lys211, Tyr214, Lys216, Met226 and Ser229, which contributed to ligand binding and had a significant effect on drug specificity, absorption and metabolism. In addition, in the complex of naringenin-4IDZ, a total of four amino acid residues, namely Lys88, Leu91, Arg96 and Lys216, formed ionic interactions with the ligand. In addition, fifteen water bridges are formed by amino acid residues, namely Arg80, Gln86, Lys88, Leu9, Arg96, Leu109, Lys211, Tyr214 and Ser229.

Interactions of the 4IDZ protein with the quercetin ligand through the involvement of amino acid residues form different types of bonds to the active center of the protein (Figure 10C). A total of ten amino acid residues, namely Gln86, Leu91, Arg96, Tyr106, Lys216, Ser229, His232, Asp233, Glu234 and Arg322 form hydrogen bonds. Among these hydrogen bonds, the value of the interaction fraction of the amino acid residues Arg96, identified as 1.5, shows that they are involved in the establishment of several contacts of the same subtype with the ligand. The hydrogen bonds with the residues reinforce the complex formed by Arg80, Val83, Arg84, Gln86, Leu90, Leu91, Arg96, Tyr106, Lys107, Tyr108, Leu109, Asn205, Ser229, Tyr214, Lys216, Ala227, Trp230, His231, His232, Asp233, Glu234, Asn235, Thr320 and Arg322. The complex formed eight hydrophobic bonds with the amino acid residues Val8, Ile85, Leu90, Pro93, Tyr108, Leu109, Val228 and His231 and four ionic bonds with the amino acid residues Arg96, Lys216, His232 and Arg322.

The complex of atropine and 4IDZ protein formed the hydrogen bonds Arg27, Gly28, Ser29, His30, Gln41, Ser56, Glu59, Gln86, Leu109, Ala159, Glu161, Lys216. Glu217, Glu218, Tyr220, Phe221, Gly222, Ser229, His232, Asp233, Glu234, Asn235, Glu250, Arg265, Asp266, Ile269, Gln291, Ala303 and Gln314. A total of five ionic bridges were formed by the amino acid residues Arg27, Glu161, Lys2`16 and Asp266. The amino acid also formed a total of 26 hydrophobic and 55 water bridges to the atropine-4IDZ complex. All bonds have a low value for the interaction fraction, but apart from this, hydrogen and hydrophobic bonds provide the strength and stability of the complex due to the maximum number of water bridges (Figure 10D).

The interaction profile of the amino acid residues with the standard drug orlistat-4IDZ complex was analysed (Figure 10E). The interaction analysis showed that compared to the complex of the four best studied compounds with the 4IDZ protein, the number of hydrogen bonds (4) formed by the amino acid residues Trp42, Gln43, Leu49 and Leu51 is very low and has fewer interaction fractions. In addition, the amino acid residues are involved in the formation of 12 hydrophobic bonds and 8 water bridges, which contribute to the stability of the complex.

**Figure 11:**
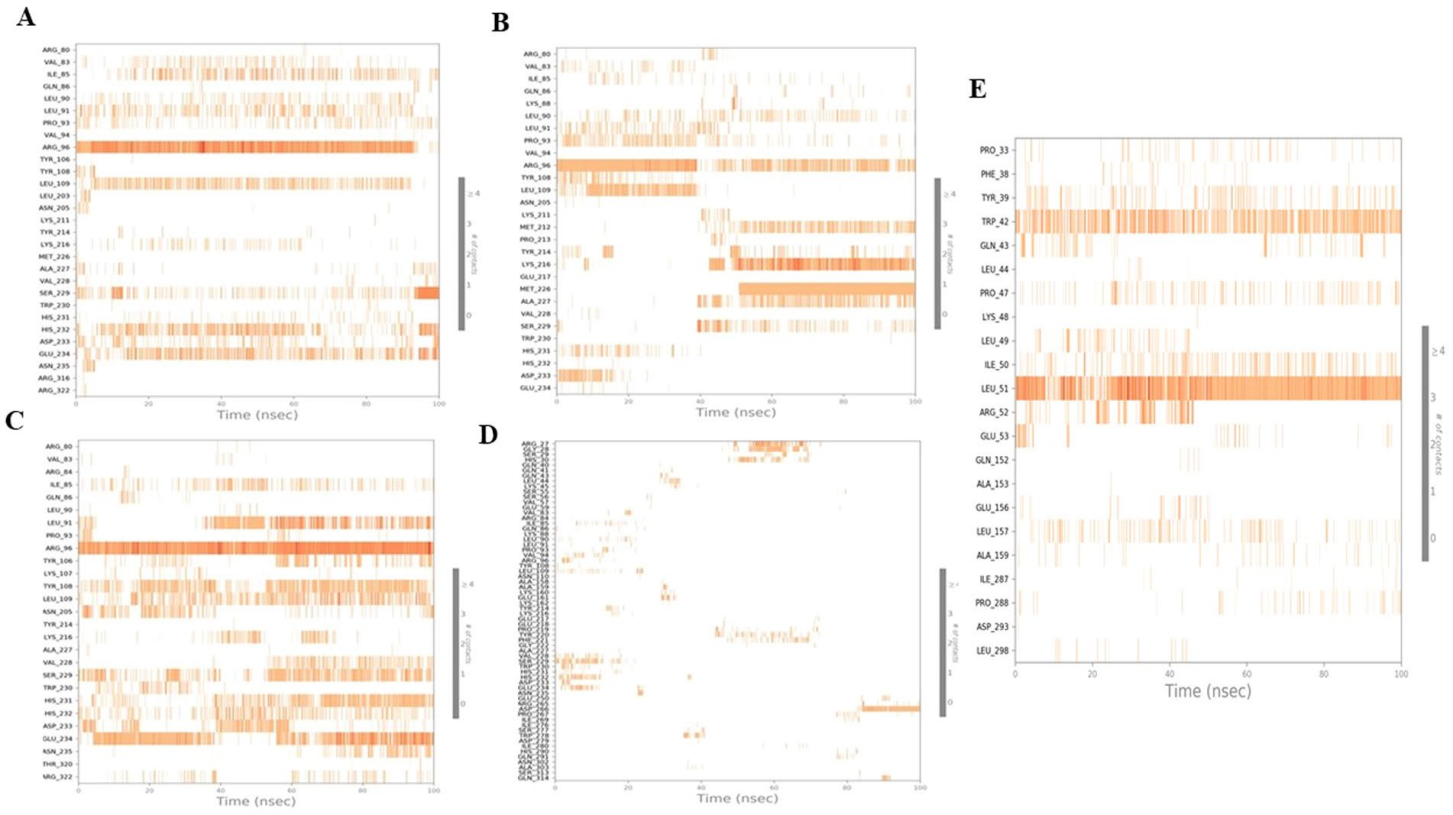
Timeline representation for 100 ns simulation run analysis of **(A)** luteolin, **(B)** naringenin, **(C)** quercetin, **(D)** atropine, and **(E)** standard drug orlistat to 4IDZ protein.

Figure 11 shows the exact interactions between the amino acid residues in the targets. The higher number of bonds in relation to the amino acid residues is represented by darker colors. The amino acid residues Arg96 and Ser229 showed strong interactions in the complex of luteolin -4IDZ. The strong interaction between the naringenin-4IDZ complex is formed by the amino acid residues Lys216 and Arg96. The strong link between the quercetin-4IDZ and the atropine-4IDZ complex is formed by six (Leu91, Arg96, Leu109, Asn205, Ser229 and Glu234) and two (Arg27 and His30) amino acid residues, respectively. The standard drug orlistat forms a strong complex with the protein with only two amino acid residues (Leu51 and Arg52), which is lower than the complex of the receptor protein with the investigated hit compounds except atropine, indicating that orlistat forms a weak binding with the target protein. The results and the data displayed in the histogram were in exact agreement

#### 3.4.4 Radius of gyration (Rg)

**Figure 12:**
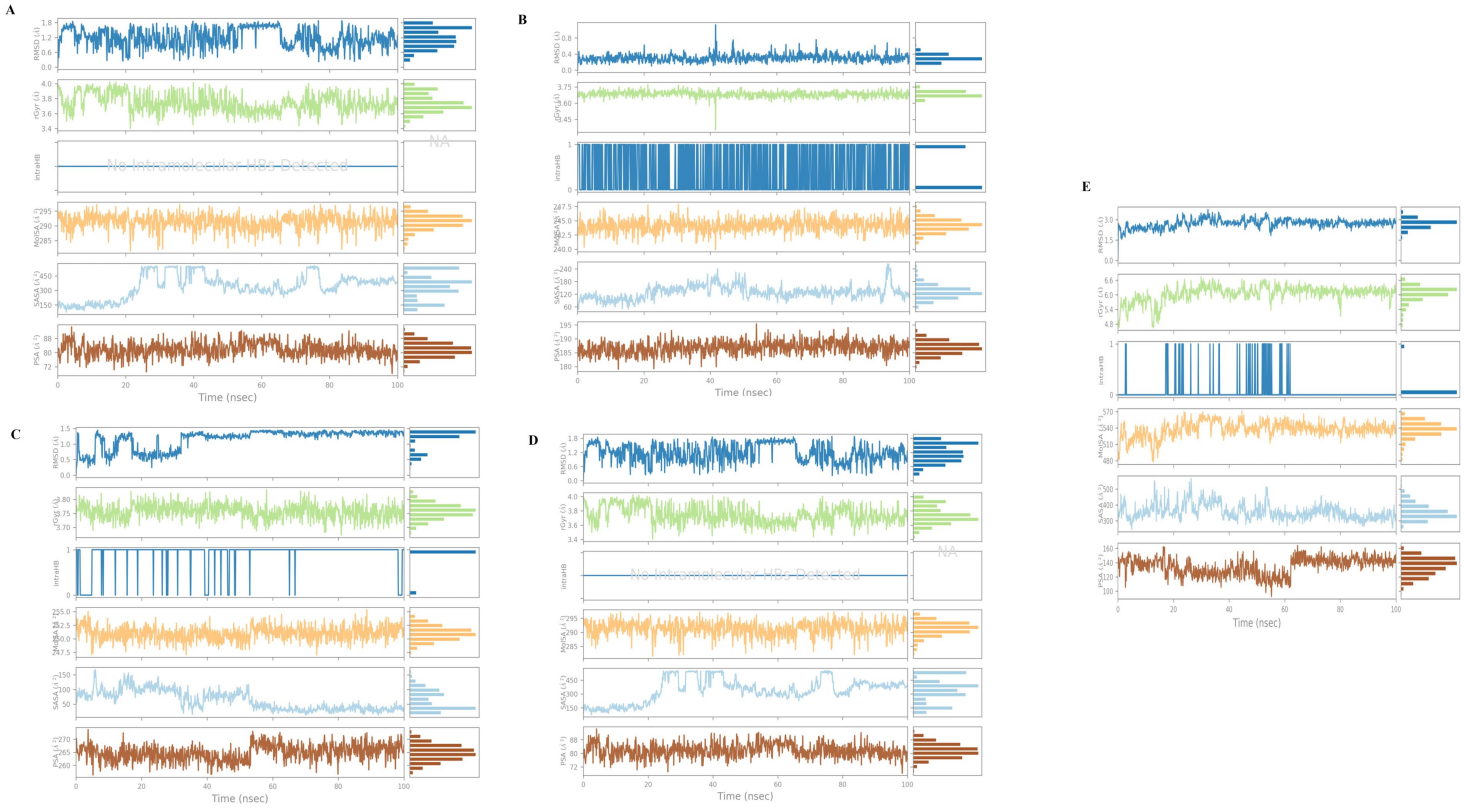
Fluctuation properties of ligands with the complexes of 4IDZ protein formed with **(A)** luteolin, **(B)** naringenin, **(C)** quercetin, **(D)** atropine, and **(E)** standard drug orlistat using radius of gyration (Rg), molecular surface area (MolSA), solvent accessible surface area (SASA), and polar surface area (PSA).

The strength and compactness of the macromolecular system is evaluated using trajectory points from MD simulations over the radius of gyration (59). According to Kumar et al. (57), Rg refers to the root mean square distance between a group of atoms and their common center of mass considering their masses. The protein-ligand system or complex may have a higher degree of conformational stability if the Rg values show less variation. The radius of gyration of the four investigated compounds and the orlistat complex with 4IDZ is represented in Figure 12. During the simulations, the protein and ligand complexes showed different patterns in their Rg values. According to the Rg profile, the protein-ligand complexes of the luteolin-4IDZ complex, the naringenin-4IDZ complex, the quercetin-4IDZ complex and the atropine-4IDZ showed an Rg value of 3.75–3.85 Å, 3.45–3.75 Å, 3.70–3.80 Å and 3.4–4.0 Å, with slight fluctuations throughout the 100 ns time. Among the four compounds studied, naringenin is very stable due to its lowest Rg value and showed minor fluctuations at 40 ns throughout the simulation period, after which this compound remains stable. In contrast to the investigated compounds, the Rg value of the standard drug (orlistat) with 4IDZ shows lower stability at 4.8–6.6 Å, which is higher than that of all investigated compounds (Figure 12).

#### 3.4.5 Solvent-accessible surface area (SASA)

The area of the ligand surface with which solvent molecules can interact is called the solvent-accessible surface area (SASA) and indicates how exposed the ligand is to its environment. The variability of the SASA can be related to how the amino acid residues are distributed either in the exposed area or in the hidden region (60). In addition, the SASA value is also useful to understand how proteins fold and maintain their stability and structure. We need to study their interactions with other proteins and surface molecules. Figure 12 shows the plot of the SASA value against the residue. The surface area of the four ligands naringenin-4IDZ complex, quercetin-4IDZ complex and atropine-4IDZ ranged from 50 to 200 A^2^, 60 to 240 A^2^, 50 to 150 A^2^ and 150 to 450 A^2^, respectively. All compounds show little fluctuation with the exception of the atropine-4IDZ complex, which shows fluctuations during a period of 20 to 80 ns and remains stable thereafter. The orlistat-4IDZ complex shows a SASA value of 300–500 A^2^, which is very high compared to the compounds studied. This discovery confirms that the complex of 4IDZ protein with the four target compounds of Little Millet studied by the docking result has more compactness and stability.

#### 3.4.6 Molecular surface area (MolSA)

Molecular surface area analysis (MolSA) was used to investigate the effects of the displacement of amino acid residues in the exposed or hidden regions by analyzing the receptor-ligand interactions. The result showed that the van der Waals surface is measured with a probe radius of 1.4 Å to determine the MolSA. An unstable protein–ligand complex is indicated by a high MolSA value, while a relatively stable complex is indicated by a low MolSA value (61). In our analysis using computer modeling based on MolSA values, we found that the complex of 4IDZ with four compounds studied is stable based on their MolSA value of 350 Å^2^. The differences between the four compounds are small, with MolSA values of 242.5 to 247.5 Å^2^, 240.0 to 247.5 Å^2^, 247.5 to 255 Å^2^ and 285 to 295 Å^2^ observed for luteolin, naringenin, quercetin and atropine, respectively. The standard drug orlistat proved to be a polar molecule and less stable than the investigated compounds, as the MolSA value of orlistat was between 480 and 570 Å2 and thus higher than that of the investigated compounds (Figure 12).

#### 3.4.7 Polar surface area (PSA)

By measuring the polar surface area (PSA) of the solvent, the ability of the ligand to bond with polar or charged solvent molecules such as oxygen and nitrogen atoms was evaluated in the simulation. During the simulation period, the ligand luteolin, naringenin, quercetin and atropine remain relatively stable (Figure 12), with a PSA value of 230 to 250 Å2, 180 to 195 Å2 and 72 to 88 Å2, respectively (Figure 12). The PSA value did not change, indicating that the ligand consistently forms stable interactions with the solvent molecules. The ligand, the standard drug orlistat, shows greater fluctuation in contrast to the other compounds. The result shows that orlistat exhibits more fluctuations during the initial period of 60 ns, with the PSA value being 100–160 after 60 ns, while orlistat remains stable throughout the simulation period of 100 ns. The fact that there were few fluctuations in the ligand properties at the beginning and middle of the simulation period indicates that the ligand was stable within the dynamic pocket of the protein.

#### 3.4.8 Torsion profile

**Figure 13:**
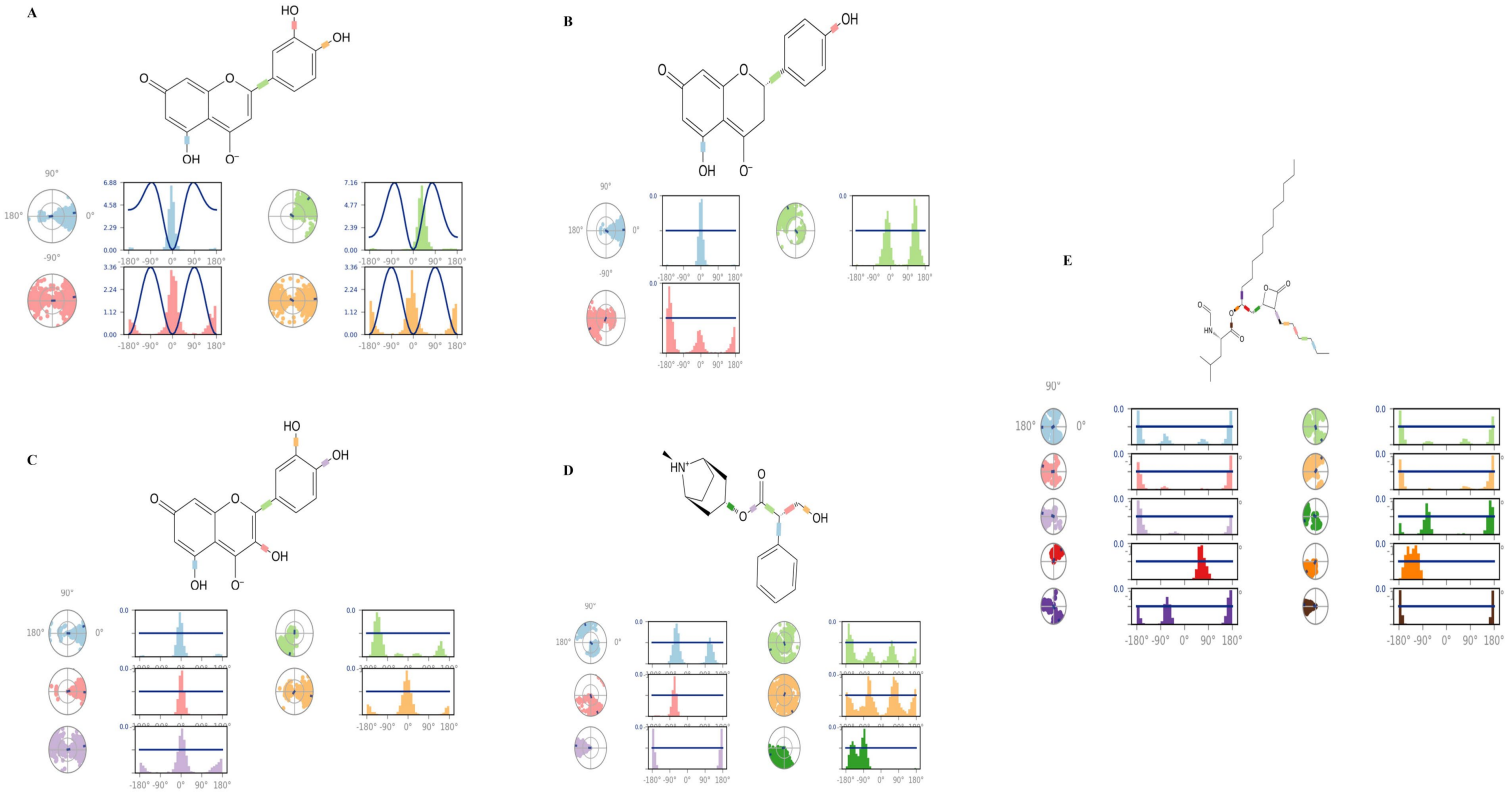
Torsional analysis of ligand-4IDZ conformations during 100 ns simulations **(A)** luteolin, **(B)** naringenin, **(C)** quercetin, **(D)** atropine, and **(E)** standard drug orlistat.

Figure 13 shows a two-dimensional schematic of the top-hit compounds and orlistat with color-coded torsional bonds. The torsional bonds of the investigated compounds as well as those of orlistat are represented by radial and colored bar plots using a uniform color scheme. Starting from the center of the radial plot, the course of the simulation is shown radially outwards. Bar charts, which provide a concise summary of the radial plots, show the probability density of the torsion angles. The rotational binding potential, measured in kcal/mol, is shown on the Y-axis of the bar graphs.

The dynamics of the torsional degrees of freedom in the ligands were analysed using the rotational binding energies. For the four compounds analysed luteolin, naringenin, quercetin and atropine the number of rotatable bonds was found to be 4, 3, 5 and 6, respectively. Luteolin in particular had four rotatable bonds between the ligand atoms 2–13, 9–10, 5–19, and 6–21. Naringenin displayed three rotatable bonds between atoms 3–12, 6–8, and 5–20, while quercetin had five between atoms 3–14, 2–12, 10–11, 6–20, and 7–22. Atropine contained six rotatable bonds between atoms 1–11, 1–13, 13–14, 14–15, 14–16, and 3–16. In contrast, the standard drug orlistat showed the highest number of rotatable bonds, with a total of ten.

### 3.5 Estimation of binding free energy

To confirm our research findings, a post-simulation study using MM-GBSA analysis was performed to predict the binding free energy of both the standard drug and the hit complexes (Table 6). This approach assesses the stability of a protein-ligand complex based on the binding energy (dGbind) released when the ligand interacts with the protein. The MM-GBSA calculations yielded binding free energies of −19.21 kcal/mol for the 4IDZ/luteolin complex, −22.75 kcal/mol for the 4IDZ/Naringenin complex, −23.98 kcal/mol for 4IDZ/Quercetin complex and − 26.86 kcal/mol for 4IDZ/atropine complex. These values were consistent with experimentally measured affinities, indicating a stronger binding affinity of four screened hits compounds to 4IDZ. In addition, standard drug orlistat exhibited highest binding affinities to 4IDZ with value of −62.17 kcal/mol.

**Table 6:** MM/GBSA profiles of screened compound and standard drug, while interacting with 4IDZ.

	dG_ Bind	_dG_Bind_ Coulomb	_Bind_ Covalent	_dG_Bind_Hbond	_Bind_Lipo	Bind_ Packing	dGvdW (Kcal/mol)
Luteolin	−19.21	−69.95	2.44	−1.42	−8.72	−1.59	−30.41
Naringenin	−22.75	−49.67	1.26	−1.53	−6.78	−0.033	−25.12
Quercetin	−23.98	−70.81	1.76	−1.89	−10.91	−3.20	−35.16
Atropine	−26.86	−4.11	0.61	−0.38	−9.53	−0.83	−21.84
Orlistat	−62.17	−11.09	3.39	−0.62	−23.24	0	−50.18

Notably, the 4IDZ/atropine complex exhibited a Coulomb energy of −4.11 kcal/mol, which differed from the other complexes, suggesting stabilization through reduced polar interactions between the ligand and receptor. The free energy of a favorable reaction is negative, and all leading ligands showed a negative dGbind. The compounds exhibited similar van der Waals interaction energies (DGvdW), suggesting their tendency to reside near the interacting amino acids of the receptor binding domain (RBD). Additionally, the negative Coulomb energy values indicate better stability of these ligands, as they do not have enough potential energy to destabilize. Table 6 provides the contributions to the total energy from various components, including hydrogen-bonding correction, lipophilic energy, pi-pi packing correction, and van der Waals energy.

## 4 Discussion

Foodomics is a burgeoning field that investigates the complex relationships between food, nutrition, and health through an omics-based approach, propelled by advances in omics technologies. Obesity, a leading contributor to various chronic diseases such as cardiovascular ailments, liver dysfunction, and diabetes (62), is a growing global health issue. It is primarily characterized by an excessive accumulation of fat in the body’s adipose tissue. Adipose tissue is categorized into two main types: white adipose tissue (WAT) and brown adipose tissue (BAT). Typically, individuals with obesity exhibit an increased proportion of WAT relative to BAT (63). Major factors contributing to obesity include poor dietary habits, particularly high-fat diets, and a sedentary lifestyle (64).

Little Millet, a prominent type of small millet, plays a crucial role in the diets of many people in developing countries due to its rich nutritional profile. It is abundant in secondary metabolites such as phenols, flavonoids, alkaloids, and terpenoids, which have demonstrated efficacy against a range of health disorders including obesity, diabetes, and cardiovascular diseases (65). The global surge in obesity can be attributed to an increased intake of high-fat, low-fiber foods, which promotes fat deposition and weight gain (66). The anti-obesity properties of Little Millet are linked to its high fiber content, resistant starch, phenols, and tryptophan—a crucial amino acid that enhances satiety and regulates appetite by modulating serotonin levels in the brain, thereby mitigating weight gain (67). Additionally, the complex starches in millet, which release glucose gradually, contribute to the regulation of blood glucose levels, thereby aiding in the management of cardiovascular diseases, diabetes, and obesity.

To heighten awareness of millets, the United Nations has designated 2023 as the “International Year of Millets,” encouraging both increased cultivation and consumption of millet products. Given its remarkable medicinal properties, incorporating millet into the regular diet could significantly assist in combating obesity. In this study, we aimed to identify the metabolites present in Little Millet grains using GC–MS and assess their potential for weight management by docking them against obesity-associated proteins. Recent research has highlighted that bioactive compounds in minor millets possess the potential to address several health disorders associated with unhealthy lifestyles, such as atherosclerosis, diabetes, and hypertension, through the inhibition of target receptor proteins (68–73).

This study represents the first comprehensive investigation into the potential of bioactive compounds in Little Millet grain extract as inhibitors of the fat mass and obesity-associated (FTO) protein. By employing molecular docking, pharmacokinetics, and dynamic simulations, we have explored the therapeutic mechanisms through which Little Millet may combat obesity. Previous research has often been limited by the scope of docking studies, either using compounds from earlier investigations or those identified through high-performance liquid chromatography (HPLC). Our study highlights the shortcomings of such approaches, which typically involve a limited number of metabolites or less detailed profiling methods. In contrast, the use of gas chromatography–mass spectrometry (GC–MS) in our study enabled us to generate a more comprehensive metabolite profile, enhancing our understanding of the range of bioactive compounds present in Little Millet and their potential therapeutic benefits.

Our results suggest that incorporating Little Millet into the diet may offer significant advantages in combating obesity—a subject that has been underexplored, particularly in industrialized countries. In the realm of Foodomics, our research has elucidated how metabolites derived from Little Millet could aid in obesity treatment by examining their interactions with the FTO protein (4IDZ). Molecular docking was employed to predict the binding affinities of these metabolites and their potential impacts on the protein’s function. Additionally, molecular dynamics simulations provided insights into the stability and behavior of these interactions over time, deepening our understanding of how these metabolites might influence obesity-related processes.

Our GC–MS analysis identified a total of 145 bioactive compounds in Little Millet, including fatty acids (e.g., 9,12-Octadecadienoic acid, n-Hexadecanoic acid, and 9-Octadecenoic acid), phenols (e.g., catechin, ferullic acid, and *β*-d-glucopyranoside), flavonoids, and alkaloids (e.g., atropine, naringenin, octodrine, luteolin, Procyanidin B1, and scopolin). Notably, compounds such as 9,12-Octadecadienoic acid and n-Hexadecanoic acid were present in substantial quantities, corroborating previous findings that highlight the health benefits of these compounds (74). Our study has also identified novel compounds, including scopolin, octodrine, luteolin, atropine, and Procyanidin B1, which were not reported in earlier studies. These unique compounds exhibit anti-inflammatory effects that could be beneficial for managing weight gain in humans.

Furthermore, our findings align with reports by Dayakar Rao et al. (42), who noted that polyunsaturated fatty acids in Little Millet, such as 9-Octadecenoic acid (868 mg) and 9,12-Octadecadienoic acid (1,320 mg), are found in much higher concentrations compared to rice (150–197 mg for oleic acid and 200–616 mg for 9,12-Octadecadienoic acid) (42). These trans fatty acids contribute to obesity reduction by decreasing inflammation, improving fat metabolism, and promoting satiety through the release of hormones like cholecystokinin and glucagon-like peptide-1 (75, 76). Additionally, sugars and sugar alcohols such as mannitol, xylitol, and myo-inositol, identified in Little Millet via GC–MS, act as low-calorie sweeteners that improve insulin sensitivity, reduce overall calorie intake, suppress appetite, and help control body mass index, thereby assisting in weight management (77, 78).

The FTO gene, recently identified as playing a significant role in obesity, impacts energy metabolism by disrupting the balance between energy and adipose tissue regulation when its activity is altered (79). Genomic studies have linked variations in the FTO gene to human obesity and metabolic disorders (80). Suppressing the FTO protein is one of many mechanisms that could mitigate the development of metabolic syndrome, including obesity (81).The overexpression of FTO may enhance fat storage and contribute to obesity, as this protein is crucial for regulating fat accumulation (82).

In our study, we evaluated the binding of four major compounds—luteolin, naringenin, quercetin, and atropine—to the FTO protein. The binding energies recorded ranged from 7.22 to 8.15 kcal/mol, which are higher than that of the standard drug orlistat (5.96 kcal/mol). The structure and function of the FTO protein are influenced by intramolecular interactions such as hydrogen and hydrophobic interactions, which are critical for maintaining the stability of the complex conformation. Luteolin, in particular, emerged as a promising phytoconstituent from Little Millet, demonstrating a strong interaction with the active site of the FTO receptor, with a binding energy of 8.15 kcal/mol, surpassing orlistat. Comparatively, Aabideen et al. (83) reported that luteolin from *Taraxacum officinale* L. acted as a potent anti-obesity agent with a binding energy of −13.9305 kcal/mol, outperforming orlistat (−9.1309 kcal/mol) (83). Luteolin modulates metabolism by attenuating pro-inflammatory cytokines such as TNF-*α* and IL-6, thereby mitigating inflammation (84). Additionally, luteolin exhibits anti-adipogenic effects by downregulating pathways crucial for adipogenesis, such as PPARγ and C/EBPα, thus preventing the formation of mature adipocytes (85).

Among the 133 bioactive compounds identified in Little Millet grains, naringenin (8.05 kcal/mol) and quercetin (7.61 kcal/mol) showed strong affinities for the FTO receptor. Naringenin forms two hydrogen bonds with the receptor, while quercetin forms only one. Previous studies corroborate the effectiveness of quercetin and naringenin in binding to the FTO protein, contributing to anti-obesity effects (79, 86, 87). Our results align with previous findings that quercetin and naringenin are more effective than orlistat in inhibiting FTO, with binding energies of −8.0 and − 7.8 kcal/mol, respectively (86). However, a slight discrepancy exists between our findings and those of Zhang et al. (87), who reported higher binding energies for quercetin (−7.87 kcal/mol) compared to naringenin (−6.21 kcal/mol) (87). This variation may be due to differences in computational methods or receptor conformations. Notably, we identified a novel compound, atropine, with a strong binding affinity for the FTO protein, showing a binding energy of −7.22 kcal/mol. This is the first study to suggest atropine’s potential role in targeting FTO for obesity treatment.

Ensuring safety and efficacy is a critical aspect of drug discovery, given that every drug carries the potential for side effects. The functionality of bioactive compounds is intricately linked to their medicinal chemistry, which governs their action within the human body. Our evaluation of the highest scoring compounds identified by YASARA focused on a comprehensive assessment of their pharmacokinetic properties, pharmaceutical capabilities, and ADMET profiles. Lipinski’s Rule of Five was applied to assess the therapeutic potential of these compounds, an essential step in drug development. Notably, all top-rated compounds met the criteria for desirable drug-like properties, characterized by adequate membrane permeability and oral bioavailability. In contrast, the standard anti-obesity drug orlistat failed to meet these criteria, with the Brenk and Pain Alerts indicating unsatisfactory drug-like properties. Orlistat also exhibited high toxicity, notably impacting liver function, and demonstrated high skin permeability and low metabolic rate, underscoring its limitations as an oral anti-obesity therapeutic. Remarkably, the compounds under investigation showed no adverse effects and robust gastrointestinal (GI) absorption without crossing the blood–brain barrier (BBB).

To identify the most promising molecule for inhibiting the 4IDZ protein, molecular dynamics simulations were conducted to analyse RMSD, protein secondary structure, radius of gyration (Rg), protein-ligand contacts, solvent-accessible surface area (SASA) energy, molecular surface area (MolSA), and polar surface area (PSA) for the top four ranked complexes, along with the orlistat-4IDZ complex. The results demonstrated that the complexes formed with the investigated hit compounds exhibited greater stability compared to the orlistat-4IDZ complex. Previous studies have shown that quercetin maintains superior stability in complex with obesity-associated proteins compared to orlistat, as indicated by RMSD and RMSF analyses (88).

These findings collectively highlight the potential of targeting the FTO protein in the development of novel anti-obesity therapies. The strong binding affinities observed for luteolin, naringenin, quercetin, and atropine suggest that these compounds, particularly those derived from natural sources such as Little Millet, hold promise as candidates for future drug development. The identification of atropine as a novel FTO inhibitor opens new research avenues, emphasizing the need for further experimental validation to evaluate its therapeutic potential in weight management and metabolic disorders. Additionally, these results provide scientific evidence supporting the health benefits of Little Millet consumption in controlling weight gain.

Further research is warranted to evaluate the anti-obesity effects of metabolites present in Little Millet through *In vitro* and *In vivo* studies using obese mice or animal models that replicate human obesity and metabolism. Furthermore, an *in vitro* digestion assay could be employed to assess the bioavailability of significant metabolites identified as potent inhibitors of the FTO protein in Little Millet grains through docking studies.

## 5 Conclusion

Obesity remains a significant global health challenge, characterized by the excessive accumulation of adipose tissue. This study utilized an *In-silico* approach to investigate substances extracted from Little Millet, focusing on their potential as anti-obesity agents. Molecular docking studies revealed that the active site of the FTO protein binds strongly with four principal compounds: luteolin, naringenin, quercetin, and atropine. These compounds exhibited superior binding affinity compared to the standard anti-obesity drug, orlistat. Notably, orlistat demonstrated limited efficacy due to its adverse side effects and toxicity. Stability simulations further indicated that the complexes formed between the four selected compounds and the FTO protein (4IDZ) were more stable and compact than those involving orlistat, which showed greater fluctuations and instability. These findings underscore the potential of the bioactive compounds in Little Millet as promising candidates for anti-obesity functional foods. However, to validate these findings, additional *In vivo* and *In vitro* research is essential. Such studies will help confirm the health benefits observed in controlled environments and living organisms. This research highlights the potential for Little Millet to gain wider acceptance, thereby improving public health and boosting the income of farmers. Additionally, the identification of high-scoring compounds from this study could inform future millet breeding programes, enhancing the nutritional value and health benefits of Little Millet.

## Data Availability

The original contributions presented in the study are included in the article/Supplementary material, further inquiries can be directed to the corresponding authors.

## Footnotes

^1^https://www.rcsb.org/

^2^https://pubchem.ncbi.nlm.nih.gov/
