# Supplementary material for: Exploring the therapeutic mechanisms of millet in obesity through molecular docking, pharmacokinetics, and dynamic simulation
Source: Front Nutr. 2024 Oct 18;11:1453819. doi: 10.3389/fnut.2024.1453819 (PMC11528469; doi:10.3389/fnut.2024.1453819)
Supplement: Supplementary file 1 [file Table_1.DOCX]

**Supplementary Table 1. Bioactive compounds present in little millet identified by GCMS**

| 10-Octadecenoic acid | Benzyl alcohol, p-hydroxy-.alpha.-[(methylamino)methyl]- |
| --- | --- |
| 14-Pentadecynoic acid | Bromosuccinate |
| 18,19-Secoyohimban-19-oic acid | Butanal |
| 2,5-Dimethoxy-4-(methylsulfonyl)amphetamine | Butane |
| Brolamfetamine | Butanedioic acid |
| Cyclohexanol, 2-(methylaminomethyl)-, trans | Butanediol |
| l-Alanine ethylamide, (S)- | Butanoic acid |
| l-Guanidinosuccinimide | Catechine |
| +/-.-Tetrahydro-3-furanmethanol |  |
| 1-Decanol, trifluoroacetate | D-Fructose |
| 1-Docosene | D-Glucitol |
| 1-Methyldodecylamine | d-Gluco-hexodialdose |
| 1,2-O-Isopropylidene-D-glucofuranose | D-Gluconic acid |
| 1,3-Propanediamine, N-methyl | D-Mannitol |
| 11-Eicosenoic acid | D-Ribofuranose |
| 11-Octadecenoic acid, methyl ester | D-Ribonic acid |
| 2-Butenedioic acid | D-Ribopyranose |
| 2-Deoxy ribose | D-Ribose |
| 2-Deoxy-galactopyranose | dl-3-Aminoisobutyric acid, N-methyl-, methyl ester |
| 2-Octenoic acid | dl-Alanine ethyl ester |
| 2-Propenoic acid | Docosanoic acid |
| 2-Propenoic acid, n-pentadecyl ester | Eicosanoic acid |
| 2,3-Dimethoxyamphetamine | Erucic acid |
| 2,3,4-Trihydroxybutyric acid | Ethanamine, N-methyl |
| 2,5-Dimethoxy-4-ethylamphetamine | Ethylamine, 2-(adamantan-1-yl)-1-methyl |
| 2(3H)-Furanone | glucitol |
| 3-Eicosene | Glucose oxime |
| 3-Ethoxy-4-hydroxymandelic acid | Glycerin |
| 3-Hydroxydodecanedioic acid | Glycidol |
| 3,4Dehydro-dl-proline | Heptadecanoic acid, heptadecyl ester |
| 4-Iodo-3-methoxyamphetamine | Heptanedioic acid |
| 9-Hexadecenoic acid | Hexanoic acid, pentadecyl ester |
| 9-Octadecanoic acid | Hexopyranose |
| 9-Octadecenamide | Hydroquinone-.β-d-glucopyranoside |
| 9-Octadecenoic acid | Inositol |
| 9,12-Octadecadienoic acid | Isosorbide |
| 9,12-Octadecadienoic acid (Z,Z)-, methyl ester | Levoglucosan |
| Acetamide | Levoglucosenone |
| Acetic acid | Malic acid |
| Actinobolin | Melibiose |
| Alpha-D-Galactoside | Mannonic acid |
| Alpha-D-Glucopyranoside | Myo-Inositol |
| Altronic acid | N-Dodecylmethylamine |
| Arabinitol | n-Hexadecanoic acid |
| Arabinonic acid | Norpseudoephedrine |
| Azelaic Acid | Octanoic acid, 8-hydroxy-, methyl ester |
| Benzeneethanamine, 2-fluoro-.beta.,5-dihydroxy-N-methyl- | Oleic Acid |
| Benzoic acid | p-Hydroxynorephedrine |
| Pentafluoropropionic acid, heptadecyl ester | Theritol |
| Pentitol | Thiodiglycol |
| Pentonic acid | trans-9-Octadecenoic acid |
| Propane | Trichloroacetic acid |
| Propanedioic acid | Trichloroacetic acid, hexadecyl ester |
| Propanenitrile | Undecylenic Acid |
| Propanoic acid | Uridine |
| Prost-13-en-1-oic acid | Xanthosine |
| Prosta-5,13-dien-1-oic acid | Xylitol |
| Pyrimidine-2,4(1H,3H)-dione | Xylonic acid |
| Ribonic acid | β-D-Galactofuranose |
| Scopolin | Paradrine |
| Atropine  Octodrine  Procyanidin B1  Cinnamic acid  Gallic acid  Norpseudoephedrine  Naringenin | Luteolin  Quercitin  Syringic acid  Ferullic acid  caffeic acid  Syringic acid  Gallic acid |
